# Breast cancer in the era of integrating “Omics” approaches

**DOI:** 10.1038/s41389-022-00393-8

**Published:** 2022-04-14

**Authors:** Claudia Rossi, Ilaria Cicalini, Maria Concetta Cufaro, Ada Consalvo, Prabin Upadhyaya, Gianluca Sala, Ivana Antonucci, Piero Del Boccio, Liborio Stuppia, Vincenzo De Laurenzi

**Affiliations:** 1 Center for Advanced Studies and Technology (CAST), “G. d’Annunzio” University of Chieti-Pescara, 66100 Chieti, Italy; 2 Department of Psychological, Health and Territory Sciences, “G. d’Annunzio” University of Chieti-Pescara, 66100 Chieti, Italy; 3 Department of Innovative Technologies in Medicine and Dentistry, “G. d’Annunzio” University of Chieti-Pescara, 66100 Chieti, Italy; 4 Department of Pharmacy, “G. d’Annunzio” University of Chieti-Pescara, 66100 Chieti, Italy; 5 Department of Medicine and Aging Science, “G. d’Annunzio” University of Chieti-Pescara, 66100 Chieti, Italy; 6 Zayed Centre for Research into Rare Disease in Children, University College London, London, WC1N1DZ UK

**Keywords:** Cancer genomics, Breast cancer

## Abstract

Worldwide, breast cancer is the leading cause of cancer-related deaths in women. Breast cancer is a heterogeneous disease characterized by different clinical outcomes in terms of pathological features, response to therapies, and long-term patient survival. Thus, the heterogeneity found in this cancer led to the concept that breast cancer is not a single disease, being very heterogeneous both at the molecular and clinical level, and rather represents a group of distinct neoplastic diseases of the breast and its cells. Indubitably, in the past decades we witnessed a significant development of innovative therapeutic approaches, including targeted and immunotherapies, leading to impressive results in terms of increased survival for breast cancer patients. However, these multimodal treatments fail to prevent recurrence and metastasis. Therefore, it is urgent to improve our understanding of breast tumor and metastasis biology. Over the past few years, high-throughput “omics” technologies through the identification of novel biomarkers and molecular profiling have shown their great potential in generating new insights in the study of breast cancer, also improving diagnosis, prognosis and prediction of response to treatment. In this review, we discuss how the implementation of “omics” strategies and their integration may lead to a better comprehension of the mechanisms underlying breast cancer. In particular, with the aim to investigate the correlation between different “omics” datasets and to define the new important key pathway and upstream regulators in breast cancer, we applied a new integrative meta-analysis method to combine the results obtained from genomics, proteomics and metabolomics approaches in different revised studies.

## Introduction

Breast cancer (BC) is the most frequently diagnosed cancer and a major health issue in women, being the leading cause of cancer-related deaths among women, worldwide [1–3]. Even if 5–10% of BC cases are due to hereditary and genetic factors, non-hereditary factors have been described as the primarily responsible for differences in incidence between countries and ethnic groups [1]. BC is recognized as a heterogeneous disease, both at the molecular and clinical level. In this regard, at the beginning of the millennium, Perou and colleagues did a groundbreaking discovery in BC using DNA microarray. Their extensive gene expression profiling found variation in expression of 1753 genes in 84 experimental samples. They concluded that BC is not a uniform disease, instead it is composed of five distinct subtypes: luminal A, luminal B, basal-like, normal breast-like, and HER-2 enriched [2–5]. In fact, these subtypes of BC are also well known as “*Perou’s molecular subtypes*” [6]. Moreover, being a multifaceted disease, BC is characterized by intratumoral and intertumoral heterogeneity. Specifically, it is defined as intratumoral when cells within a tumor in a single patient are involved, while intertumoral when cells of the same subgroup of tumors in different patients are involved [2]. Thus, the heterogeneity found among BCs led to the concept that BC is not a single disease and rather represents a group of distinct neoplastic diseases of the breast and its cells [7]. Nowadays, therapeutic options for BC treatment include surgery, radiotherapy, chemotherapy, and targeted therapies [4, 8]. Despite recent and important advances in understanding BC biology, diagnosis and treatment, several significant clinical issues still remain unclear. In particular, these unmet clinical needs are related to prevention, diagnosis, tumor progression, treatment, therapeutic resistance and metastasis formation [3, 9]. In this context, modern systems biology based on “omics” approaches can potentially make a major contribution to overcome these problems. In fact, in the era of precision medicine, “omics” strategies and their integration in the study of BC may be considered as a new biomarker discovery tool, leading to novel biomarker molecules and molecular signature with a potential in clinical practice [9]. It is worth noting that a molecular profiling is involved in BC, as in any phenotypic alterations, and is recognizable on different levels: genome, transcriptome, proteome, and metabolome. At the beginning of the twenty-first century, revolutionary progress of high-yield and innovative technologies in nucleic acid sequencing and mass spectrometry (MS) have driven the advent of Genomics, Transcriptomics (functional genomics), Proteomics and finally of Metabolomics, leading to the “multi-omics” era [9–11]. Anyway, we should consider that, even if each “omics” approach is essential to systems biology, giving its contribution in a specific way to shape the biological phenotype under study, some are more mature than others [11]. These “omics” approaches are quite different from the conventional methods for the study of BC complex biology, mainly for the possibility of obtaining a huge number of molecular measurements within cells, a tissue or in biological fluids [12]. Once applied to a pathological condition of interest, they allow to obtain a snapshot of the underlying biology, with a resolution never achieved before. Interestingly, the application of each aforementioned “omics” technology and their integration by network science for studying BC gives the possibility of holistic investigation and contextually of a comprehensive pathophysiologic understanding of such a complex disease, with the promise of providing novel insights into precise diagnosis, potential therapeutic options and tailored treatment [11, 13, 14]. Thus, the main advantage of “omics” strategies is to bring out the omics-based molecular profiling with the clinical outcome under study. Actually, in the multi-omics context it has become evident that the use of integrative tools and of computational approaches is necessary [14] to deeply understand biological mechanisms from a system-wide perspective [15].

The present manuscript reviewed recent works in literature of the findings by omics-based studies in BC until 2020. In particular, we summarized and updated previously published literature on BC molecular candidates obtained by the application of three kinds of omics approaches, including genomics, proteomics, and metabolomics. In addition, we applied a network science paradigm performing a systematic integration of the molecular alterations at multiple levels including genome, proteome, and metabolome. In fact, elaborating heterogeneous-omics data sets has the potential to gain novel, mechanistically significant insights into the BC disease [14, 16].

Finally, we aimed to prove that the real challenge of multi-omics investigations lies in the integration of their frameworks and the cautious interpretation of the myriad of data in order to gain further insights on BC and to move toward P4 medicine (preventive, predictive, personalized, and participatory) [11, 14].

## Genomics in BC

The history of BC genomics can be broadly divided into two categories, before next-generation sequencing (NGS), or pre-NGS, and after NGS, or post-NGS. Pre-NGS era is mainly characterized by studying of individual genes associated with BC. During the pre-NGS era, the hallmark genes such as BRCA1 and BRCA 2 were discovered. After the advent of NGS, the study of BC genomics boomed, and BC study was not limited to only few genes. A number of new genes and intergenic interactions were discovered during post-NGS era of BC. The genes associated with BC can be found on Supplementary Table S1. The genes are categorized in different groups, based on the type of alteration (i.e., mutation/polymorphism) or susceptibility of developing cancer (high/moderate/low penetrance). Gene expression or transcriptomic data are not included in this review.

### Pre-NGS era of BC

Genomics is one of the important factors determining the outcome of BC. In the ’90 s, researchers observed that family history is the strongest single predictor of a woman’s chance of getting BC. After a long search, two genes, BRCA1, was discovered in 1994, and the second, BRCA2, in 1995, was found to be associated with the BC in women [17, 18]. Importantly, a meta-analysis shows that the mean cumulative BC risks at the age of 70 were 57% for BRCA1 and 49% for BRCA2 mutation carriers [19]. The search for other genes continues and several genes are known to be involved in somatic and inherited susceptibility to BC. Apart from BRCA1 and BRCA2 genes, which are used as the gold standard for genetic testing for BC, there are several other genes involved in varying extent in the susceptibility of BC. The pieces of evidence at that time made two things clear: first, BC is not a single disease, but instead, it is composed of a spectrum of tumor subtypes with distinct molecular, cellular and somatic changes [20]. Secondly, various rare genetic syndromes are linked with increased BC risk. For example, mutations that inactivate the Tp53 gene, which primarily causes Li–Fraumeni syndrome, are also associated with increased susceptibility to BC [21, 22]. The risk of developing BC before the age of 45 is 18-fold higher with the females affected with Tp53 mutation as compared to the general population [21]. Germline mutations in the Tp53 gene have been estimated to account for <1% of BC cases [23–25]. However, somatic mutations in the Tp53 gene are reported in 19–57% of human BCs [26–28]. Inactivation of just one allele for Tp53 gene may be sufficient for BC development [28]. Likewise, mutations in the ATM gene are responsible for Ataxia-telangiectasia (A-T) disease. Though A-T patients do not survive to an age at which BC generally occurs [29] A-T carriers (heterozygous for ATM mutations) appear to have an increased BC risk [30–33], with an estimated increased risk of 11% by the age of 50 and 30% by the age of 70 [34]. A study of 138 Austrian hereditary breast and ovarian cancer patients without BRCA1 and BRCA2 mutations showed functionally significant ATM germline mutations in at least 8.7% of the patients [35]. The penetrance for one of the mutations (L1420F) was estimated to be 85% at age 60. Renwick et al. sequenced ATM in 443 BRCA-negative cases from families with at least three BC-affected members and in 521 controls. Nine truncating and exon-skipping mutations were identified in cases, while only two were found in controls. All mutations found in cases were predicted to cause AT, and seven had been observed previously in AT cases [36]. Bernstein et al. performed an ATM mutation screen in 708 unilateral BC survivors who developed contralateral BC following radiotherapy and 1397 who did not. They found that women with AT-associated ATM mutations treated previously with radiation had a significantly greater risk of contralateral BC than unexposed women either with no mutation or unexposed women with the same mutation [37]. Similarly, a mutation in the PTEN gene leads to the Cowden syndrome in 80% of Cowden syndrome families [38, 39]. On the other hand, the same truncating PTEN mutations in Cowden syndrome families are associated with 25–50% lifetime BC risk in women [39–41]. Loss of heterozygosity (LOH) at the PTEN locus is found in 11–41% of sporadic BCs [42–45]. In one study (in 177 BC patients with a positive family history for BC and without BRCA1 and BRCA2 mutations), an association was found between a polymorphism in intron 4 of the PTEN gene and a lower age of diagnosis of BC (42.7 versus 48.1 years) [46]. Moreover, Peutz–Jeghers syndrome is an autosomal dominant disorder, caused by truncating germline mutations in the LKB1 gene [47, 48]. Patients with Peutz–Jeghers syndrome have an increased BC risk [47–49]. Another genetic disorder, Neurofibromatosis type 1 (NF1) is a common autosomal dominant disorder associated with an increased risk for neoplasms [50]. Women with NF1 develop BC at younger ages than the general population, with an average of <50 years old. Moreover, the risk of developing BC wit NF is 6.5-fold higher in women aged 30–39 years and 4.4 times higher among women aged 40–49 [51]. In patients with no BRCA1 or BRCA2 mutations, LOH in the NF1 region was found to be responsible for the onset of BC [52].

### Post-NGS era of BC

NGS of DNA introduced rapid and cost-efficient way to identify genes involved in BC [53]. NGS allows detecting multiple genetic alterations at the same time, using the same assay, leading to the concept of “multigene sequencing”. Based on the multigene sequencing, several other genes were found to be involved in the susceptibility of the BC. BRCA1-associated ring domain (BARD1), a direct interacting partner of BRCA1, is likely to be a low–moderate penetrance BC risk gene [54]. Loss-of-function (LoF) mutation in BRAD1 gene was present in 0.51% of BC patients. Also, BARD1-mutated BC patients showed a significantly younger mean age at first diagnosis (42.3 years, range 24–60 years) compared with the overall study sample (48.6 years, range 17–92 years) [55]. Similarly, germline LoF mutations in BRCA1 interacting protein C-terminal helicase 1 (BRIP1), which is a low penetrance gene, are associated to contribute to BC risk, particularly among patients who develop the disease at an early age [56]. Normal BRIP1 activity is required for DNA interstrand cross-link (ICL) repair and is thus central to the maintenance of genome stability. Next-generation sequencing of germline DNA in 2,160 early-onset BC and 1,199 patients with ovarian cancer revealed nearly 2% of patients carry a very rare missense variant in BRIP1, which is 3-fold higher than the frequency of all rare BRIP1 missense alleles reported in more than 60,000 individuals of the general population [56]. The study by Seal et al. sequenced the exons and exon-intron boundaries of BRIP1 in 1212 BC cases with a family history of the disease and no BRCA mutation and 2081 controls and found mutations in nine cases (0.74%) but only in two controls (0.10%) [57]. Similarly, mutated CHEK2 or CHEK2 pathogenic variant (PV) is a high penetrance BC gene [58]. Biallelic CHEK2 PV carriers have a higher risk for BC, are more likely to be diagnosed younger, and have multiple primary BCs compared to monoallelic carriers [58]. A population-based study found that deletion in CHEK2 (CHEK2*1100delC) is present at a frequency of 1.1% in controls, 5.1% in cases with a family history, and 13.5% in cases with a family history of male BC in a population with a positive history of BC but no BRCA mutation [59]. SMAD4 is another gene that gets inactivated in BC patients. SMAD4 is a common signal transducer in the bone morphogenetic protein (BMP)/transforming growth factor-β (TGF-β) signaling pathway, and functions as a transcription corepressor for human estrogen receptor α (ERα) [60]. SMAD4 is located on 18q21, a region frequently lost in BCs [61]. Inactivation or suppressed expression of TGF-β/SMAD4 signaling has been found to play an important role in BC development [61–63].

Besides the above-mentioned genes, NGS revealed that germline LoF mutations in PALB2 confers a predisposition to BC. PALB2 interacts with BRCA1 and BRCA2, and biallelic mutations in PALB2 (also known as FANCN), disrupts the Fanconi anemia–DNA repair pathway and increases BC predisposition [64, 65]. Furthermore, L35Pa, a pathogenic missense mutation in PALB2, abrogates the PALB2-BRCA1 interaction which may lead to failure in BC suppression [66]. In recent years, RAD51C and RAD51D are the other two genes that have been used in the screening of BC susceptibility [67, 68]. The estimated cumulative risks of developing BC to 80 years is 21% for RAD51C and 20% for RAD51D pathogenic variant carriers. BC risks for RAD51C and RAD51D pathogenic variants could be 44–46%, for carriers with two first-degree relatives diagnosed with BC [69]. Other notable genes that get mutated in BC are NBN and CDK12.

NBN gene mutation shows moderate to low penetrance [70]. Among NBN variants, a protein-truncating variant, c.657del5, is sufficiently common in some Eastern European populations to allow its evaluation in case-control studies. A meta-analysis of 10 studies reported strong evidence of an association with BC risk for this variant [34, 70].

Moreover, CDK12 (cyclin-dependent kinase 12), a low penetrance gene, is a regulatory kinase with evolutionarily conserved roles in modulating transcription elongation. In BCs, CDK12 is also frequently co-amplified with the HER2 oncogene [71]. CDK12 expression was found to be high in 21% of primary unselected BCs [72, 73]. Other low penetrance genes that are mutated in BC include MutYH, MSH2, CDKN2A and APC. The MutYH gene is involved in base excision repair. Carriers of variants in MutYH, although not very common, may have an increased risk of BC [74]. In a study in Italy by Rizzolo et al., biallelic MutYH pathogenic variants (p.Tyr179Cys/p.Arg241Trp) in one MBC patient with a phenotypic manifestation of adenomatous polyposis and Monoallelic pathogenic variants in 14 (2.5%) MBC patients were identified. Overall, the study suggests that MutYH pathogenic variants may have a role in MBC and, in particular, the p.Tyr179Cys variant may be a low/moderate penetrance risk allele for MBC [75]. On the other hand, Thibodeau et al. identified two patients with BC, each carrying a pathogenic germline MutYH variant with a somatic MutYH copy loss leading to the germline variant being homozygous in the tumor [76]. Regarding MSH2, a study showed that 1.1% woman with BC carries MSH2 mutation [77]. Moreover, another low penetrance gene CDKN2A mutation was identified (A148T variant) in 157 of 3,069 women with BC (5.1%) in a study in Poland. Their study shows that CDKN2A A148T variant seems to contribute to early-onset BC [78]. Furthermore, the adenomatous polyposis coli (APC) gene is a regulatory gene of the Wnt/β-catenin signaling pathway, which are independently involved in maintaining low levels of β-catenin in the cell. In an Indian study, a single nucleotide polymorphism (SNP), rs2229992 was identified in APC gene, with an increased risk of breast carcinogenesis in a BC and control population from eastern India [79].

## Proteomics in BC

In recent years, omics approaches have emerged as a promising and extremely useful tool to reveal innovative molecular pathways as well as to identify and quantify the levels of molecules differentially expressed. In this scenario, mass spectrometry (MS) techniques have occupied an increasingly central position in the investigation of potential biomarkers, applied above all to complex and multifactorial pathologies, such as the study of cancer. To date, several studies based on the quantification of proteins are carried out with approaches based on the use of antibodies which are strongly linked to the availability, quantity, affinity and specificity [80]. Furthermore, as their use is inevitably linked to a starting hypothesis, this could hinder the study of the “neglected proteome” for the study of new potential biomarkers, or new biological pathways and functions related to BC [80, 81]. Proteomics can be defined as a high-throughput and large-scale study of proteins, investigating their classification, expression levels, properties and function [82]. Proteomic approaches based on MS techniques can be classified into two macro-groups: targeted and non-targeted proteomics. The main objective of the non-targeted applications is to cover almost complete proteomic knowledge, suitable for the application of the biomarkers discovery. The targeted approach, on the other hand, is more suitable for the validation of the results obtained from the first “discovery” approach and therefore for clinical applications. This fit-for-purpose approach has the aim of maximizing the coverage of potential objectives that can be assessed in the early stages for the discovery of biomarkers or therapeutic targets [80].

### Proteomics features and studies in BC

In this literature review, to deepen proteomics features and studies in BC, we searched the PubMed site including quite recent scientific works approximately from 2010 to 2020, typing the keywords “Breast cancer proteomics Biomakers” and including in this study only the proteomics works followed by validation of the results obtained using different approaches. The research for BC is strongly aimed at the study of diagnosis, prognosis and disease course biomarkers in easily accessible biofluids, for this reason we listed in Supplementary Table S2 the candidate BC biomarkers found in serum [83–97], plasma [98–107], urine [108, 109], and Nipple Discharge biofluids [110].

In particular, human urine is considered one of the most interesting biofluids as it represents an excellent resource for the discovery of new biomarkers, with the advantage over tissue biopsy samples thanks to the ease and the less-invasive nature of the collection [111]. Furthermore, the high level of stability, the ease of sampling and an inactive and low complexity test matrix offer numerous potential advantages also compared to the use of other biofluids such as serum and plasma [112].

Interestingly, nipple secretion has also been proposed as a new clinical diagnostic technique and source of secreted proteomes that may reflect early pathological changes in the ductal-lobular epithelial microenvironment and could therefore provide specific BC biomarkers, while remaining an easily accessible and non-invasive source [110].

In recent years, much attention has been paid not only to the study of cell markers, but also especially to that of the “secretome”. “Secretome” is defined as the rich and complex set of molecules and proteins secreted by living cells and released from the surface. The need to develop increasingly effective cancer biomarkers has shifted the focus towards the study of tumor cell secretome as a means of identifying and characterizing diagnostic and prognostic markers and potential pharmacological and therapeutic targets, bearing in mind that secretome proteins carry out a key role in cell signaling, communication and migration [113].

Of note, recent technological developments in the field of proteomics have significantly stimulated and facilitated research in this direction. Hence, in this Review, the works involved in the research of protein biomarkers of BC through proteomics approaches on secretome and on extracellular matrices were collected, schematizing for each biomarker the clinical significance associated (Table S3).

As mentioned above, BC is considered a heterogeneous disease and the most common mistake is to treat BC as a single entity. Current insights from studies on intratumoral heterogeneity and cancer stem cells increase the possibility that multiple BC subtypes can coexist within a tumor and, therefore, the stratification of tumors is fundamental to obtain better clinical results [114].

In this work, we report a list of proteins considered Cell/Tissue Breast Cancer Biomarker, through proteomics studies and subsequent validation experiments (Table S4). Most of the research works cited in Table S4 are studies based on the research of protein biomarkers closely related to the onset of metastases or tumor growth and progression. Other proteins listed in the tables have been studied according to their correlation to epithelial–mesenchymal transition [115–117].

BC cell lines have been widely used for BC modeling, which includes a group of diseases with distinct phenotypic associations. The wide use of cell lines in biomarker research is due to its extremely homogeneous and potentially unlimited content for proteomics studies. Moreover, BC cell lines are also relatively easy to culture.

Numerous studies on protein biomarkers of prognosis, tumor growth and aggression have been conducted on various cellular subtypes of BC. Most of the proteomic studies reported on cellular models are exclusively conducted on Triple-negative (TN) BC (TNBC) tumor subtypes, any BC characterized by the lack of expression of estrogen and progesterone receptor, and of human epidermal growth factor receptor 2. In Table S4, asterisk (*) and hash (#) symbols are used to highlight the potential protein biomarkers found on the cell subtypes or on primary tumor tissues of BC patients, respectively.

As a group, TNBCs is viewed clinically as an aggressive subgroup of BC with a complex and heterogeneous genomic landscape, with an earlier age of presentation and requiring adjuvant chemotherapy to improve survival. The classification of TNBCs in subtypes on the basis of gene expression patterns can provide benefits from specific therapeutic agents [118].

In consideration of complex genomes, high levels of genetic instability, and a high degree of intertumor and intratumor heterogeneity, conventionally TNBCs are defined high-grade carcinomas.

Most TNBCs defined as high-grade tumors have an unfavorable prognosis. Anyway, a subset of TNBCs, comprising histologically low-grade lesions and therefore defined low-grade TNBCs, vastly differs from those high-grade TNBCs and has a favorable outcome. High-grade TNBC include carcinomas with apocrine differentiation, carcinomas with bone marrow characteristics and metaplastic breast carcinomas [118]. Furthermore, current studies suggest several subgroups of low-grade TN malignancies such as a subset of lesions that includes microglandular adenosis, atypical microglandular adenosis and acinic cell carcinoma. Low-grade variants of metaplastic breast carcinomas and solid papillary carcinoma with polarity reversal are additional rare special histological types of low-grade TNBC. The complexity and study of the various histological subtypes of TN disease should not be overlooked, as therapeutic approaches for rare low-grade TNBC subtypes are fundamentally different from those of high-grade TNBC [118].

However, BC cell lines are known to develop mutations during initial establishment and subsequent culture series [118]. In fact, BC cell lines are extremely useful, but often considered rough models for tumors of the same subtype.

The onset of metastasis is one of the most important factors causing the death of patients with BC. In fact, the detection of metastases from BC is an indication of tumor aggression, and if detected early, it should facilitate the correct management of the progression of BC. Therefore, it is very important to look for effective biomarkers for the metastasis and prognosis of BC.

Numerous studies focusing on the identification of metastasis-related factors, potentially used as prognostic markers related to tumor size, axillary lymph node status and histological grade / subtype, have been found [119–122]. The profiling of tumor tissue proteomics provides important information on the discovery of biomarkers [123, 124]. In Table S4 the protein markers obtained from studies conducted and validated in primary tumor tissues of BC patients have been highlighted with the symbol “#”.

In addition, in recent years many researchers have shifted their attention from the study of BC cell lines to cancer steam cells (CSCs). As a result, some protein biomarkers placed on the surface of the CSCs [125] or involved in self-renewal of CSCs [126] have been identified, indicated with the symbol “§“ in Table S4. CSCs are known to play an important role in the recurrence of cancer in almost 65% of cases [127, 128]. Unlike cancer cells, CSCs are quiescent, resulting resistant to anti-cancer drugs. Furthermore, after anti-cancer treatment, these cells can become active and multiply rapidly [129, 130].

For this reason, it has been necessary to develop specific CSC tracking techniques and markers in order to maximize the therapeutic effect of the treatment in cancer cells.

## Metabolomics in BC

Metabolomics, one of the newest promising techniques in the “omics” field, allows the quantification of metabolites and/or the evaluation of their ratios in a biofluid, cell, tissue, organ or organism at a given state. As one of the most recent members of the omics family, there has been significant progress in metabolomics in the last decade, primarily driven by technological advances in MS. The metabolome is dynamic, so that metabolite levels and/or ratios can result altered in a pathological condition, thus highlighting abnormal metabolic functions, mainly in complex diseases as BC [11]. Moreover, variations in the metabolome may be the result of genetic, environmental factors, as well as exogenous and endogenous factors [131]. Nowadays, it has become clear that metabolomics, through the comprehensive and quantitative analysis of low-molecular-weight compounds in a system provides the clue to a phenotype, with the potential for a great clinical impact [12, 132, 133]. In fact, even a comprehensive understanding of the state of genes, transcripts, and proteins in a living system is not sufficient to reveal its phenotype [132, 133]. When combining metabolomics with genomics, possibly transcriptomics, and proteomics, a complete understanding of biological mechanisms from a system-wide perspective can be provided [7]. It is now well accepted the idea that metabolites represent the link between genotype and phenotype, and that the study of metabolome offers a significant advantage, allowing to highlight the end-point markers of biological events [133]. Unlike genomics and proteomics, metabolomics is able to provide evidence of end-point markers for diagnosis or evaluation of response to therapy [133]. In this view, the transcripts deriving from DNA are translated into proteins, enzymes necessary for the catalysis of metabolic intermediates [7]. In fact, metabolites, identified by metabolomics strategies either in a targeted or in unbiased manner, are downstream and thus are more sensitive signs of alterations in biological system [131, 134]. Anyway, we should recognize that metabolomics is still emerging with the potential to be deeply (highly) effective in the discovery of molecular candidates for cancer diagnosis, prognosis and treatment[135].

There are two main strategies for metabolic studies: targeted and untargeted analysis of endogenous and exogenous metabolites (<1500 Da) in biological samples at a given point of time. Targeted analysis aims to the quantitative measurement of predetermined compounds taking part in the same biochemical pathway. Thus, the metabolic profile characteristic of that sample might be altered as a result of a gene mutation, diet, drugs, or environmental factors [136]. Non-targeted metabolomics may be described as an open analysis not driven by any preliminary hypothesis for the comprehensive determination of all metabolites present in a sample, with the aim to define alterations in whole metabolome as metabolic fingerprinting characterizing the biological system under specific conditions [131, 136]. Even if metabolomics enables high-throughput analysis of different metabolic pathways and processes all at once, it should be emphasized that it is not yet possible to analyze the entire metabolome and that no single analytical platform can describe all the possible metabolites present in a complex sample, because of their chemical differences and concentration [11]. However, it should be remembered that a wide coverage of metabolism can be obtained by combining two high resolution analytical frameworks: MS, coupled with different separation techniques, and nuclear magnetic resonance (NMR) spectroscopy [137]. In both platforms for metabolomics investigations, after data acquisition, statistical analysis is crucial to give the right value and significance to the dataset previously obtained by the analytical tools [131]. NMR and MS are the most popular platforms for metabolomics and are complementary to each other, even if each approach has advantages and limitations [134]. NMR, the pioneering platform in metabolomics, requires no or low sample pretreatment and allows for reproducible, non-destructive and non-selective analysis, also enabling the simultaneous measurement of different classes of metabolites. Such approach generates a NMR spectrum providing structural information for metabolite identification. Anyway, it presents lower sensitivity if compared with MS [9, 131, 136]. MS is an increasingly used analytical tool for metabolomics applications aiming at the identification of potential biomarkers in different clinical fields. In general, direct-injection MS analysis allows to obtain metabolic profile or fingerprint, but this approach also has some limitations in terms of co-suppression and low ionization efficiency. For this reason, MS is often coupled to a separation technique, based on gas chromatography (GC-MS), liquid chromatography (LC-MS) or capillary electrophoresis. Briefly, GC-MS analysis are characterized by high specificity, sensitivity and accuracy, but they have limitations in mass range (mass-to-charge ratio, *m*/*z* 30–550) and in some requirements since the compounds of interest need to be volatile and also thermo stable. The overmentioned requirements are not necessary in LC-MS analysis, the most promising and widely used tool for metabolomics in clinical applications [131]. The growing use of LC-MS can be explained with its high-throughput, soft ionization, and with the possibility to cover a wide range of metabolites. The success and popularity of LC-MS-based metabolomic study is essentially due to the versatility dependent on the sample pretreatment, more simple and rapid in comparison to GC-MS technique, and to the variety of separation possibilities and mass analyzer [131, 138].

### Metabolomics features and studies in BC

Bringing our attention back to BC and considering that cancer is a disease that contributes to alterations in cellular metabolism, metabolomics-based studies in the area of BC may be an useful tool for novel biomarker discovery, identification of the related disturbed pathway, early diagnosis, and the evaluation of treatments [131]. When mentioning the perturbed pathway and fighting BC, knowledge on metabolism is highly important [139]. In 1924, Otto Warburg put forward his metabolic hypothesis for cancer. In oncology, the term Warburg effect indicates cancer dependence on fermentative glycolysis, even when oxygen supply is adequate [140]. Therefore, in cancer tissues, the metabolic state often reflects hypoxic metabolism [139]. Metabolomics studies have also described an altered protein and lipid metabolism in cancer [141]. One hypothesis is that even small tumors influence the way metabolites are used in the whole organism. Several metabolic changes have been observed in the blood or urine which reflect one further step downstream in metabolic transformation. Samples for the metabolomic analysis of the BC include urine, serum, plasma, saliva, or tissue and, since metabolites are end products of cellular processes, their concentrations reflect the systems-level response of biological systems and may be valuable for diagnostic tests and therapeutic interventions.

Metabolomics is based on recently developed technologies that allow the quantitative investigation of a multitude of different metabolites. A comprehensive coverage of metabolism can be achieved only by a combination of analytical approaches. The most popular approaches for metabolomics involve GC-MS, LC-MS or NMR spectroscopy. MS-based approaches are typically more sensitive. NMR spectroscopy can be applied to intact tissue samples and even to observe metabolites in vivo, with the technology being referred to as magnetic resonance spectroscopy in the clinic. An improvement of NMR spectroscopic procedure is a technique called high resolution magic angle spinning (HR-MAS) NMR spectroscopy, which involves spinning of a biopsy sample at an angle to the magnetic field, to improve the spectrum resolution. GC-MS-based analyses of metabolic impact or changes in metabolism have a long history in BC research—for example, analysis of phospholipids [142], pharmacology (including tamoxifen metabolism) [143], exposure to xenobiotics [144], estrogen levels [145] or urinary metabolomic profiles [146, 147].

In this work, we report a list of metabolites considered Biofluids/Cell/Tissue Breast Cancer Biomarker, obtained using different approaches (Tables S5 and S6).

NMR studies of human BC samples [148] have found higher contents of Gly, Tau, Lact, and Succ, and lower levels of Gluc and inositol for tumors compared to noninvolved breast tissue. In addition, lipidomics studies have showed that the lipidomics profile correlates with cancer tissue and tumor grade. One of the other profound changes that accompany tumor proliferation is alteration in the proportion of choline-containing metabolites. A series of studies has provided a comprehensive picture of altered Cho metabolism in tissues, as shown in the table [149, 150]. Numerous studies have showed raised levels of choline and its phosphorylated metabolites in subjects with benign and malignant tumors only, and these metabolites have been used for classifying tumor types during the immortalization of cell lines and during apoptosis and necrosis. Choline, phosphocholine and glycerophosphocholine can be observed in clinical magnetic resonance spectroscopy. This has a considerable clinical potential, especially as the HR-NMR analysis is fast, relatively inexpensive, and nondestructive.

In recent years, much attention has been paid to the study of metabolite markers in biofluids.

Although both NMR and MS are commonly used for urine metabolomics, most BC studies were based on MS. Urine samples have the information that can discriminate between normal and BC groups. Urine BC study identified among metabolites as potential biomarkers, amino acids, organic acids, and nucleosides including dimethylarginine, tyrosine, phenylalanine, pantothenic acid, succinyladenosine, dimethylguanosine, apronal, threonyl carbamoyl adenosine, tryptophan, kynurenic acid, nico-tinuric acid, and indolelactic acid [151]. Homovanillate, 4-hydroxy-phenylacetate, 5-hydroxyindoleacetate and urea have been identified as biomarkers for BC from urine using GC-MS [146, 152].

The most easily identifiable clinical biomarkers are derived from blood. Therefore, it is an essential question whether the metabolic response observed in the blood is directly derived from tumor tissue, or whether it represents a more general response of the organism to the presence of a tumor. Several studies identified markers associated with amino acid metabolism, glycolysis, and fatty acid metabolism. Biomarkers reported for metastatic subjects include high values of Phe, Gluc, Pro, Lys, and *N*-acetyl-Cys [153, 154], and low values of lipids. The final products of β-oxidation (Acac and 3-HB) and lipid degradation (Gluc), *N*-acetyl glycoproteins (NAC 1 and 2), Pyr, Glut and mannose have been reported from the analysis of serum of early and advanced BC patients. Overall reoccurring marker metabolites include His, Pro, Phe, Glu, 3-HB, Lact, and lipids [155]. Thus, several critical pathways for the early diagnosis of BC have been discovered, including the metabolism of taurine and hypotaurin, and the metabolism of alanine, aspartate, and glutamate [153, 156, 157]. Wang et al. [158] used a dried blood spot approach for rapid BC detection. In this study, the target analytes were 23 aminoacids and 26 acylcarnitines, and based on the results piperamide, asparagine, proline, tetradecenoylcarnitine/palmitoylcarnitine, phenylalanine/tyrosine, and glycine/alanine could be used as potential biomarkers to diagnose BC.

Another explored biological fluid is saliva and from metabolites identified, 3-methyl-pentanoic acid, 4-methyl-pentanoic acid, phenol, p-tert-butyl-phenol, acetic, propanoic, benzoic acids, 1,2-decanediol, 2-decanone, and decanal seem to be relevant for the discrimination of BC patients [159]. Another type of molecules, the polyamines, including N-acetylated forms, are associated with tumor growth due to their biosynthesis and accumulation [160].

In literature, the reports performed involving human cell lines focus mainly on diagnostic purposes. Finally, in the volatile composition (VOMs) of BC cell lines, 2-pentanone, 2-heptanone, 3-methyl-3-buten-1-ol, ethyl acetate, ethyl propanoate, and 2-methyl butanoate were detected only in cultured BC cell lines [161]. These VOMs are formed endogenously or obtained from exogenous sources (e.g., environmental, lifestyle, biological agents), and can be recognized as a useful tool to BC non-invasive diagnosis.

### Data processing and elaboration

In this review, in order to combine the results from different “omics” studies, we introduced a new methodical framework by performing a meta-analysis through data “omics” integration.

In particular, we used Ingenuity Pathway Analysis software (IPA, Qiagen, Hilden, Germany) for “Core Analysis” to map statistically each gene or protein or metabolite for their functional annotation, such as network discovery, Upstream Regulator Analysis (URA) and downstream effects networks. Details of data processing and elaboration by IPA are fully described in Supplementary Materials.

### “Omics” integration in BC

Following the revision of recent works in literature of the findings by omics-based studies in BC until 2020, we focalized our attention on BC molecular candidates obtained by the application of three specific omics approaches including genomics, proteomics and metabolomics, providing a systematic and detailed integration of the molecular alterations at multiple levels to better describe the pathological phenotype of such a complex disease.

We have discussed above each corresponding “omics” technique as used in the processing of biological data, starting from genomics, the oldest of the “omics” technologies, for DNA, and going on with proteomics for proteins and finally with metabolomics for metabolites [9]. Thus, the term “omics” means an approach capable of generating a complete data set of something measurable [133]. For sure, two are the most important tools which allowed “omics” approaches to reveal their great potential: NGS and MS [135]. Anyway, it should be recognized that statistical and bioinformatics tools are necessary for the processing of the large amount of data turning out by the use of such “omics” approaches [9].

The meta-analysis we conducted on the IPA tool showed good agreement with the literature currently available especially considering the Proteomics and Metabolomics approaches in BC. As shown in Table 1 the “Disease and Functions” that the data uploaded on the IPA described are fully related to the topic and, above all, they showed functions up-regulated (in orange) and down-regulated (in blue) often in agreement considering the dataset of Proteomics and Metabolomics individually.

**Table 1:** Disease and biofunctions resulted from the meta-analysis on IPA tool for Genomics, Proteomics, and Metabolomics single data sets, respectively.

**Fig. 1:**
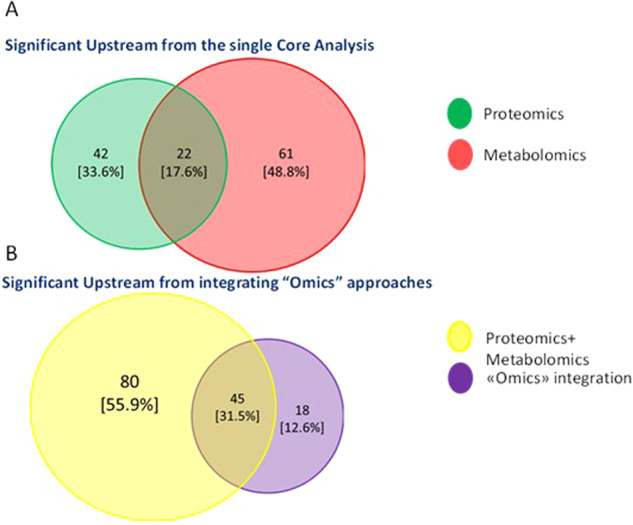
Upstream Regulator Analysis Results. **A** Venn diagram for significant upstream (both activated and inhibited) from the single “Core Analysis” using IPA tool based on Proteomics (in green), or Metabolomics (in light red). **B** Venn Diagram for significant upstream (both activated and inhibited) from integrating “Omics” approaches (in violet) vs the sum of the significant upstream obtained by each single approach (Proteomics + Metabolomics, in yellow).

**Fig. 2:**
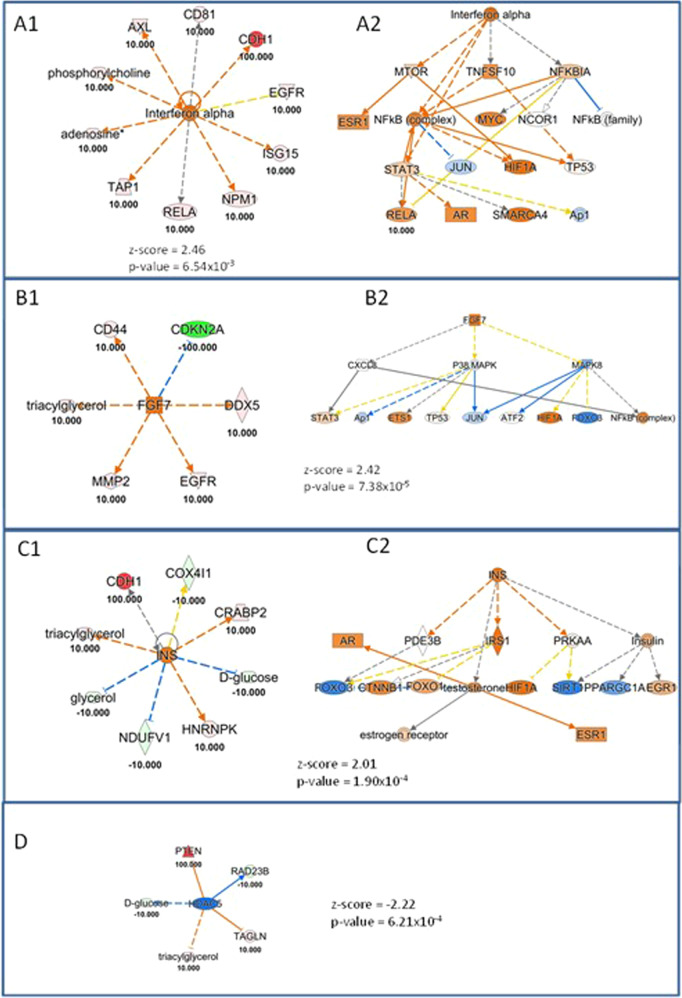
Upstream Regulator Analysis, based on “omics” integration using the Ingenuity Pathway Analysis software. Orange and blue shapes represent predicted activation or inhibition, respectively. The predicted relationship between genes may lead to direct activation (orange solid lines) or direct inhibition (blue solid lines). Red and green color indicate genes, proteins, and metabolites increased and decreased in expression, respectively, while the numbers represent the measurements of their expression. **A1** shows the proteins and metabolites of the loaded dataset involved in the activation of the upstream regulator Interferon alpha. **A2** shows the mechanistic network, theoretically reconstructed that underlies the activation of the upstream Interferon alpha. **B1** shows the proteins and metabolites of the loaded dataset involved in the activation of the upstream regulator Fibroblast growth factor 7 (*FGF7*). **B2** shows the mechanistic network, theoretically reconstructed that underlies the activation of the upstream *FGF7*. **C1** shows the proteins and metabolites of the loaded dataset involved in the activation of the upstream regulator Insulin (*INS*). **C2** shows the mechanistic network, theoretically reconstructed that underlies the activation of the upstream *INS*. **D** shows the proteins and metabolites of the loaded dataset involved in the down-regulation of the upstream regulator Histone deacetylase 5 (*HDAC5*).

Moreover, we found upstream regulators resulting from first loading of the datasets individually and subsequently from the integration of protein and metabolic biomarker candidates much interesting. As shown by the Venn diagram in Fig. 1A, 17.6% of significantly regulated upstream are in common considering the Proteomics and Metabolomics datasets individually. When we put together all the data collected from the literature (protein and metabolic candidate biomarkers), 18 new upstream results were significant in the meta-analysis on IPA, demonstrating the enormous potential that an integrated omics approach can generate (Fig. 1B). Among these interesting upstream regulators (highlighted by data integration and listed in Table 2), we decided to discuss and deepen the most interesting ones: Histone deacetylase 5 (*HDAC5*), Insulin (*INS*), Fibroblast growth factor 7 (*FGF7*) and Interferon alpha. For each upstream regulator considered, we have plotted a network showing which genes, metabolites, and proteins of the loaded dataset were considered in identifying the aforementioned upstream (Fig. 2A1–C1, D). Interestingly, Fig. 2A2–C2 show the theoretical networks that the activation or inhibition of the aforesaid upstream would contribute within the biological system, as a sort of mechanistic prediction. In particular, a panel of 10 biomarker candidates including proteins and metabolites (Fig. 2A1) demonstrated an up-regulation of Interferon alpha, in agreement with the literature, especially in aggressive cell lines [162]. In fact, in agreement with literature data, increased levels of interferon alpha have been reported in inflammatory BC, the most aggressive and lethal subtype of BC. Another interesting upstream is *FGF7* (Fig. 2B1, B2) which is also up-regulated and is known as a regulator involved in tumor growth and invasion not only in BC but also in breast and ovarian cancer [163, 164]. As reported in Fig. 2C1, the simultaneous modulation of some metabolites, such as D-Glucose, Glycerol and Triacylglycerols together with 5 proteins *COX4I1*, *CRABP2*, *HNRNPK*, *NDUFV1*, and *CDH1* promotes an up-regulation of *INS. INS* is a known upstream regulator involved in tumorigenesis through a direct effect on epithelial tissues or indirectly by affecting the levels of other modulators, such as the family of insulin-like growth factor (*IGF*) receptors, sex hormones and adipokines [165]. This can also be highlighted from the mechanistic network in Fig. 2C2.

**Table 2:** List of significant upstream results only from the integration of Proteomics and Metabolomics data.

The four upstream systems deemed most interesting and discussed in more detail are identified in bold.

Finally, *HDAC5* was significantly inhibited, as shown in Fig. 2 Panel D. Indeed, it is often down-regulated or eliminated in human cancers, such as prostate cancer [166]. Conversely, the elevated expression of *SOX9* and *HDAC5* is associated with lower survival rates in BC patients treated with tamoxifen. *HDAC5* was widely expressed in human BC tissues and high *HDAC5* expression was associated with a lower prognosis, while *HDAC5* knockdown inhibited cell proliferation, migration, invasion and enhanced apoptosis [167].

## Conclusion

As well know, adding “omics” to a molecular term connotes a comprehensive evaluation of a set of molecules, and thus multi-omics approaches through high-throughput technologies give the possibility to understand the flow of information underlying a disease, from the original cause of disease to the functional consequences [168], also leading to a crucial change in clinical research [169]. Importantly, if the analysis of data from a single omics technology is limited to correlations and mainly reflects reactive processes rather than causative ones, the integration of data from multi-omics approaches is often applied to explain potential causative changes that lead to disease, or the therapeutic targets. When applying a multi-omics strategy to a disease, it is important to consider the nature of the disorder: simple disease or complex disease. In fact, the etiology of a multifaceted disease as BC is much more complicated and is not focused on a single specific factor but rather on a combination of different factors [169]. In this review, we applied a new integrative meta-analysis method to combine the results obtained from different revised studies. Our meta-analysis proved to be a powerful tool not only to investigate and summarize the correlation between different “omics” datasets, but also to distinguish and highlight new important key pathways and upstream regulators related to BC. Hopefully, the results obtained by our speculation suggest that an in-depth description of the pathological phenotype in BC could be only reached by a proper integration of the large number of biological components, their complex interactions, and their relationships with environment. Therefore, in a systems biology view, data integration is necessary for the comprehensive understanding of the wide dataset arising from multi-omics approaches in the study of a complex disease, as BC. In fact, it is important to emphasized that BC, as each biological phenomenon, is characterized by interdependent layers of biological features. While a single omics approach can catch only a slice of the complex pathological system, multi-omics integration offers an unprecedented opportunity that is the possibility of capturing a deeper and more complete description of the pathological phenomena under study, with translation into clinically relevant information [11, 14, 170]. In conclusion, in comparison to traditional analysis focused on single biological layer, integrative and holistic multi-omics approaches, despite being complicated by the high dimensionality and heterogeneity of the data and the lack of universal analysis protocols, represent new opportunities for studying complex diseases in a more comprehensive way [171–173].

## Supplementary information

Supplementary Materials

## Data Availability

All data needed to evaluate the conclusions in the paper are present in the paper and/or the Supplementary Materials. Additional data and information related to this paper may be requested from the authors.
