# Supplementary material for: Breast cancer in the era of integrating “Omics” approaches
Source: Oncogenesis. 2022 Apr 14;11(1):17. doi: 10.1038/s41389-022-00393-8 (PMC9010455; doi:10.1038/s41389-022-00393-8)
Supplement: Supplementary file 1 — Supplementary Materials [file 41389_2022_393_MOESM1_ESM.docx]

Supplementary Materials

Review

Breast Cancer in the era of integrating “Omics” approaches.

Claudia Rossi^1,2^*, Ilaria Cicalini^1,3^, Maria Concetta Cufaro^1,4^, Ada Consalvo^1,3^, Prabin Upadhyaya^5,6^, Gianluca Sala^1,3^, Ivana Antonucci^1,2^, Piero Del Boccio^1,4^, Liborio Stuppia^1,2^, and Vincenzo De Laurenzi^1,3^

^1^ Center for Advanced Studies and Technology (CAST), “G. d’Annunzio” University of Chieti-Pescara,

66100 Chieti, Italy

^2^ Department of Psychological, Health and Territory Sciences, “G. d’Annunzio” University of Chieti-Pescara, 66100 Chieti, Italy

^3^ Department of Innovative Technologies in Medicine and Dentistry, “G. d’Annunzio” University of Chieti-Pescara, 66100 Chieti, Italy

^4^ Department of Pharmacy, “G. d’Annunzio” University of Chieti-Pescara, 66100 Chieti, Italy

^5^ Department of Medicine and Aging Science, “G. d’Annunzio” University of Chieti-Pescara, 66100 Chieti, Italy

^6^ Zayed Centre for Research into Rare Disease in Children, University College London, WC1N1DZ, London UK

***** Correspondence: [claudia.rossi@unich.it](mailto:claudia.rossi@unich.it) ; Tel.: +39-0871-541333

**Table S1: Genes involved in BC.** Genes are categorized as their change and penetrance. A remark has been put for each gene for their specific characteristic described in the literature.

| **Gene** | **Change** | **Gene penetrance** | **Remark** | **Reference** |
| --- | --- | --- | --- | --- |
| BRCA1 | Mutation | High | Breast cancer risk at the age of 70 is 57% for BRCA1 mutation carriers | (1-3) |
| BRCA2 | Mutation | High | Breast cancer risk at the age of 70 is 49% for BRCA2 mutation carriers | (1-3) |
| Tp53 | Inactivating Mutation | High | Tp53 mutation also causes Li-Fraumeni syndrome | (4, 5) |
| PTEN | Truncating mutation | High | Mutated in Cowden syndrome families. 25-50% lifetime BC risk in women. | (6-10) |
| BARD1 | LOF mutation | High | BARD1-mutated BC patients showed a significantly younger mean age at first diagnosis | (11, 12) |
| CHEK2 | Mutation | High | Deletion in CHEK2 (CHEK2*1100delC) | (13-16) |
| CDH1 | Germline mutation | High | CDH1 have been associated with an increased risk of hereditary diffused gastric cancer | (17-19) |
| ATM | Truncating and exon-skipping mutations | High-moderate | A-T patients do not survive to an age at which BC generally occurs. The penetrance for L1420F mutation is high (85% at age 60) | (20-26) |
| NF1 | LOH | High-moderate | Women with NF1 develop BC at younger ages | (27-29) |
| STK11/ LKB1 | Truncating germline mutation | High-Moderate | LKB1 gene is mutated in patients with Peutz-Jeghers syndrome | (30-33) |
| PALB2 | Biallelic mutations | High-Moderate | L35Pa is a pathogenic missense mutation in PALB2 | (34-37) |
| BRIP1 | LOF mutations | Moderate | Increases developing risk of BC at an early age | (38, 39) |
| RAD51C | Mutation/pathogenic variant | Moderate-Low | Risk increases with variant carriers with two first-degree relatives diagnosed with BC | (40, 41) |
| RAD51D | Mutation/pathogenic variant | Moderate-Low | Risk increases with variant carriers with two first-degree relatives diagnosed with BC | (41, 42) |
| SMAD4 | Inactivation | Moderate-low | SMAD4 is located on 18q21, a region frequently lost in breast cancers | (43-46) |
| NBN | Mutation | Moderate-low | A protein-truncating variant, c.657del5, is sufficiently common in some Eastern European populations | (47, 48) |
| MutYH | Mutation | low | p.Tyr179Cys/p.Arg241Trp are pathogenic variants of MutYH | (49-51) |
| CDK12 | Mutation | Low | The penetrance estimates of 39% by age 80 years is a cumulative risk in the absence of other causes of cancer/mortality | (14, 16, 52) |
| MSH2 | Mutation | Low | 1.1% woman with BC carries MSH2 mutation | (17) |
| APC | Mutation/polymorphism | Low | A single nucleotide polymorphism (SNP), rs2229992 was identified in the APC gene, with an increased risk of breast carcinogenesis | (53, 54) |
| CDKN2A | Mutation | Low | Variant A148T was identified in 5.1% of women with breast cancer, in a Polish study | (55) |

**Table S2: Biofluids Breast Cancer Proteomics Biomarker**

| **Biomarkers** | **UniProt/Kegg Code** | **Trend** | **Biofluid** | **Significance** | **Ref** |
| --- | --- | --- | --- | --- | --- |
| HDAC9 | Q9UKV0/9734 | up | serum | higher levels in the serum of recurrent breast cancer patients | (56) |
| HDAC5 | Q9UQL6/3066 | up | serum |  |  |
| SUMO1 | P63165/7341 | up | serum |  |  |
| HDAC7 | Q8WUI4/51564 | up | serum |  |  |
| OLFM4 | Q6UX06/- | up | serum | OLFM4 levels are significantly higher in the **DCIS** cohort and in the breast cancer cohort compared with the healthy controls. | (57) |
| RETN | Q9HD89/56729 | down | serum | Overexpression resistin in BC may be anindicator of improved prognosis, so down regulation of resistin may be considered as negative prognosis biomarkers | (58) |
| KCC2A | Q9UQM7/- | up | plasma | significantly higher plasma CAMK2A level in patients with metastatic TNBC with progression disease phase | (59) |
| BCAS3 | Q9H6U6/- | up | plasma | high levels in BC plasma samples | (60) |
| IRX1 | P78414/- | up | plasma |  |  |
| IRX4 | P78413/- | up | plasma |  |  |
| IRX5 | P78411/ | up | plasma |  |  |
| APOA2 | P02652/336 | up | plasma | High levels were detected and correlated with early and advanced stages of the disease | (61) |
| APOC3 | P02656/1071 | up | plasma | High levels were detected and correlated with early and intermediate stages of the disease | (61) |
| CLUS | P10909 | up  down  up | plasma  urine  serum | High levels were detected and correlated with early and intermediate stages of the disease | (61-63) |
| ZA2G | P25311/- | up | urine | candidate markers that could discriminate breast cancer patients from healthy controls | (63) |
| A2GL | P02750/- | up | urine |  |  |
| ANXA1 | P04083/301 | up | urine |  |  |
| GELS | P06396/- | down  up | urine/  plasma | candidate markers that could discriminate breast cancer patients from healthy controls | (63, 64) |
| A1AT | P01009/5665 | down  up | Urine/  serum | candidate markers that could discriminate breast cancer patients from healthy controls | (63, 65) |
| CAH2 | P00918/- | up | Nipple Discharge | protein levels differed significantly between patients with and without breast cancer | (66) |
| CATA | P04040/- | up | Nipple Discharge |  |  |
| PRDX2 | P32119/7001 | up | Nipple Discharge |  |  |
| PRG4 | Q92954/64748 | up | serum | the ratio of serum proteoglycan 4 to protease C1 inhibitor may be used for screening of early breast cancer | (67) |
| IC1 | P05155/- | down | serum |  |  |
| ECM1 | Q16610/1893 | up | urine | significantly different in breast cancer patients compared to the normal control subjects | (68) |
| MAST4 | O15021/375449 | up | urine |  |  |
| FILA | P20930/- | up | urine |  |  |
| NCHL1 | O00533/- | up | plasma | signature for breast cancer diagnosis | (69) |
| APOC1 | P02654/- | down | Plasma/  serum | signature for breast cancer diagnosis | (69-71) |
| CAH1 | P20507/8893 | up | plasma | signature for breast cancer diagnosis | (69) |
| AHSG | P02765/197 | up | serum | serum autoantibodies against AHSG protein may be useful as serum biomarkers for early-stage breast cancer | (72, 73) |
| ANGT | P01019/- | up | serum | ATII serum levels were increased in women who exhibited worse mortality outcomes | (74) |
| ANXA3 | P12429/3326 | up | serum | ANX A3 was significantly upregulated in the benign disease group sera | (75) |
| DCD | P81605/- | up | plasma | DCD levels may increase in early carcinogenesis | (76) |
| HEPC | P81172/- | up | plasma | Protein plasma levels may be of clinical usefulness to predict malignant and benign disease with respect to healthy controls | (77) |
| FRIL | P02792/- | up | plasma |  |  |
| CO3 | P01024/- | up | serum | Candidate biomarkers in serum samples from BC comparing to healthy subjects | (70, 71, 73) |
| TTHY | P02766/- | up | serum | Candidate biomarkers in serum samples from BC comparing to healthy subjects | (70) |
| GRHL3 | Q8TE85/57822 | up | plasma | Over-expression of GRHL3 in breast cancer patients in the early stages | (78) |
| TNFA | P01375/7124 | up | plasma | Over-expression in breast cancer patients in the advanced stages | (78) |
| PMS2 | P54278/5395 | up | plasma |  |  |
| CD14 | P08571/929 | up | serum | CD14 could be related to the risk of recurrence | (79) |
| TSP1 | P07996/- | up | plasma | Proteins resulted increase in breast cancer plasma | (80) |
| BRWD3 | Q6RI45 | up | plasma |  |  |
| CADH5 | P33151/- | up | serum | serum cadherin-5 discriminated patients with recurrent breast cancer from those with no sign of recurrence | (81) |
| ORM2 | Q06144/5005 | up | serum | Candidate serum biomarkers | (62) |
| VTDB | P02774/- | down | serum |  |  |
| APOA1 | P02647/- | down | serum |  |  |
| LEG3 | P17931/ | up | serum | significantly increased reactivity in early-stage breast cancer | (82) |
| RACK1 | P63244/10399 | up | serum |  |  |
| EGFR | P00533/1956 | up | plasma | validated as a predictor of breast cancer risk | (83) |

**Table S3: Extracellular matrix/Secretome Breast Cancer Proteomics Biomarker**

| **Biomarkers** | **UniProt/KeggCode** | **Trend** | **Significance** | **Ref** |
| --- | --- | --- | --- | --- |
| SERPINB1 | P30740/1992 | up | protein elevated in brain metastases | (84) |
| MFAP5 | Q13361/8076 | up | MFAP5 is secreted by CAFs,and drives the invasion and migration of MCF7 breast cancer cells. | (85) |
| TOP1 | P11387/7150 | up | Protein increased in human BRCA1 secretome | (86) |
| CDH3 | P22223/1001 | up |  |  |
| EDIL3 | O43854/- | up | In patients with metastatic breast cancer, the levels of EDIL3 on circulating extracellular vesicles are significantly elevated. | (87) |
| LTBP3 | Q9NS15/4054 | up | High extracellular matrix expression of proteins correlates with poor outcomes for breast cancer patients. | (88) |
| SNED1 | Q8TER0/- | up |  |  |
| ANXA2 | P07355/302 | up | secretion of annexin II correlated well with the invasive phenotype of triple-negative human breast carcinoma cells. | (89) |
| THS7A | Q9UPZ6/ - | up | high abundance of these proteins from cell lines and in their culture media was considered breast cancer-specific potential biomarkers | (90) |
| LG3BP(LGALS3BP) | Q08380/- | up |  |  |
| CATD | P07339/13033 | up |  |  |
| VIME | P08670/- | up |  |  |
| ZA2G | P25311/- | up |  |  |
| CD44 | P16070/960 | up |  |  |
| EGFR | P00533/1956 | up |  |  |
| IL18 | Q14116/3606 | up |  |  |

**Table S4:** **Cell/Tissue Breast Cancer Proteomics Biomarkers**

| **Biomarkers** | **UniProt/Kegg Code** | **Trend** | **Significance** | **Ref** |
| --- | --- | --- | --- | --- |
| CTSD | P07339/1509 | up | CTSD expression is associated with poor prognosis in breast cancer patients | (91) |
| RNF144A # | P50876/9781 | down | RNF144A was downregulated in a subset of primary breast tumors | (92) |
| HSPA2 | P54652/3306 | up | oncoprotein frequently upregulated in human cancer and promotes tumor growth and progression | (92) |
| CEAM6 § | P40199/- | up | novel biomarker on the cancer stem cells surface | (93) |
| AXL/UFO * | P30530 | up | specific proteome expression for basal B subclass, the more aggressive TNBC cell lines | (94) |
| PEAK1 * | Q9H792 | up |  |  |
| TGFR2 * | P37173 | up |  |  |
| SEPR * | Q12884 | up |  |  |
| UCHL1* | P09936 | up |  |  |
| MMP2 * | P08253 | up |  |  |
| MMP14 * | P50281 | up |  |  |
| LEF1 * | Q9UJU2 | up | LEF1 facilitates metastasis during breast cancer cells colonization of the brain parenchyma | (95) |
| STMN1 | P16949 | up | STMN1 was related to tumourgrade, proliferation, vascular and immune responses | (96-98) |
| UBP47 | Q96K76 | up | USP47 has a role in cell growth and genome integrity, and was correlated to Epithelial to Mesenchymal Transition | (99) |
| EPCAM # | P16422 | up | proteins were associated with the progression of breast cancer | (98) |
| FADD # | Q13158 | up |  |  |
| NDRG1 # | Q92597 | down |  |  |
| CRYAB # | P02511 | down |  |  |
| ISG15 | P05161 | up | possible biomarkers related with CNS metastasis development | (100) |
| TSP1 | P07996 | up |  |  |
| AP1M1 | Q9BXS5 | up | possible biomarkers related with CNS metastasis development | (100, 101) |
| GPDA # | P21695 | down | proteins were down-regulated in tissue tumor groups in comparison to controls | (102) |
| MGLL # | Q99685 | down |  |  |
| DOCK4 # | Q8N1I0 | up | high DOCK4 in early breast cancer is associated with aggressiveness and with future bone metastasis | (103, 104) |
| FABP7 | O15540 | up | increased levels of FABP7 correlate with a lower survival and higher incidence of brain metastases in breast cancer patients | (105) |
| DSC1 | Q08554 | up | DSC1 is validated as a protein connected with lymph node status of luminal A breast cancer and tumor grade in 96 primary breast tumors tissue | (106) |
| COMT * | P21964 | up | catechol-O-methyltransferase is successfully verified as a protein associated with lymph node metastasis of triple negative breast cancer as well as with tumor grade | (106) |
| DNAJB4 | Q9UDY4 | up | protein expression increased on Epithelial to Mesenchymal Transition induction | (107) |
| CD81 | P60033 | up |  |  |
| MUC1 | P15941 | up | upregulation of MUC in response to epidermal growth factor receptor (EGFR)-targeting treatments in breast cancer models | (108) |
| STAU1 * | O95793 | up | modulation of these proteins was noted in 231BR relative to precursor 231 cell lines | (109) |
| AT1B3 * | P54709 | up |  |  |
| NPM * | P06748 | up |  |  |
| HNRPQ * | O60506 | up |  |  |
| HNRPK * | P61978 | up |  |  |
| TUBB4B * | P68371 | down |  |  |
| TUBB5 * | P99024 | down |  |  |
| K2C1 | P04264 | up | KRT1 as the target receptor highly expressed on MCF-7 breast cancer cells | (110) |
| COF1 # | Q03048 | up | associations of protein levels with tumor grade (G3vsG1) | (97, 111) |
| PAIRB # | Q8NC51 | up | associations of protein levels with tumor grade (G3vsG1) | (97) |
| TSP2 # | P35442 | down |  |  |
| POSTN # | Q15063 | down |  |  |
| TAP1 * | Q03518 | up | candidate prognostic biomarkers in TNBC and basal-like breast cancer patients | (112) |
| CETN3 | O15182 | down | connected loss of CETN3 and SKP1 to elevated expression of epidermal growth factor receptor (EGFR) | (113) |
| SPK1 * | P63208 | down |  |  |
| TAGL # | Q01995 | up | transgelin can relate to the higher risk of metastasis development | (114) |
| TAGL2 # | P37802 | up | transgelin-2 levels were increased in metastatic and poorly differentiated tumors | (114) |
| SAP3 | P17900 | up | candidate diagnostic and prognostic marker in Breast Cancer | (115) |
| CBPB1 | P15086 | up | up-regulation of putative biomarkers in lymph node positive (versus negative) luminal A tumors | (116) |
| PDLI2 | Q96JY6 | up |  |  |
| TF65 | Q04206 | up |  |  |
| BAG6 | P46379 | Up | candidate putative biomarkers in breast cancer cell lines | (117) |
| DX39A | O00148 | Up |  |  |
| ANXA8 | P13928 | down |  |  |
| COX41 | P13073 | down |  |  |
| MMP1 * | P03956 | up | the major protein changes in 231-BR was able to predict the occurrence of brain metastases, and their potential value as therapeutic targets | (118) |
| EFNB1 * | P98172 | up |  |  |
| STML1 * | Q9UBI4 | up |  |  |
| MYCT1 * | Q8N699 | up |  |  |
| TGM2 * | P21980 | down |  |  |
| S10A4 * | P26447 | down |  |  |
| PLSL * | P13796 | down |  |  |
| NDUV1 * | P49821 | down | complex I deficiency is a potential and important biomarker of aggressiveness of human breast cancer cells | (119) |
| RAB1B * | Q9H0U4 | down | down-regulated in highly metastatic breast cancer cells | (120) |
| CAPS1 # | Q9ULU8 | up | protein is involved in processes such as calcium (Ca^(2+)^) signaling, metabolism, epithelial mesenchymal transition, metastasis and invasion | (121) |
| NB5R3 | P00387 | up | significant correlation between high CYB5R3 expression and poor disease-free and overall survival | (122) |
| IF4A1 | P60842 | up | increased protein expression is related to the metastatic phenotype of advanced breast cancer | (123) |
| EF2 | P13639 | up |  |  |
| LAMP2 | P13473 | up | potential adaptive mechanism to grow in acidic conditions | (124) |
| GDIR2 | P52566 | up | overexpression of GDIR2 in distinct breast cancer subtypes, as well as in metastatic cell lines derived from lung, prostate, and breast cancer | (125) |
| RD23B | P54727 | down | modulated in highly invasive breast cancer cell lines | (126) |
| FRIH * # §  FRIL* # | P02794  P02792 | Up  up | proteins modulated in good-prognosis patients in the training set | (101, 127, 128) |
| KCY # | P30085 | up | proteins modulated in good-prognosis patients in the training set | (101) |
| AIFM1 # | O95831 | up |  |  |
| EMAL4 # | Q9HC35 | up |  |  |
| GANAB # | Q14697 | up |  |  |
| CTNA1 # | P35221 | up |  |  |
| AP1G1 # | O43747 | up |  |  |
| STX12 # | Q86Y82 | up |  |  |
| CAPZB # | P47756 | up |  |  |
| C1TC # | P11586 | down |  |  |
| CD44 * | P16070 | up | MDA-MB-231cell surface markers | (90, 129, 130) |
| EGFR * | P00533 | up | MDA-MB-231cell surface markers | (90, 130) |
| LEG3 * | P17931 | up | MDA-MB-231cell surface markers | (130, 131) |
| BCAM * | P50895 | up | MDA-MB-231cell surface markers | (130) |
| HG2A * | P04233 | up | CD74 is overexpressed in human cancer | (132, 133) |
| CALR3 # | Q96L12 | up | overexpression of calreticulin in infiltrating ductal breast carcinomas | (134, 135) |
| LGUL # | Q04760 | up | enzyme involved in detoxification of methylglyoxal, a cytotoxic product of glycolysis in tumor breast tissues | (136) |
| IDHL | P48735 | up | candidate prognostic markers for overall breast cancer survival | (137) |
| CRABP2 | P29373 | up |  |  |
| S14L2 | O76054 | down |  |  |
| CATD | P07339 | up | high abundance of this protein from cell lines and in their culture media was considered breast cancer-specific potential biomarkers | (90, 138) |
| THS7A | Q9UPZ6 | up | high abundance of this protein from cell lines and in their culture media was considered breast cancer-specific potential biomarkers | (90) |
| LG3BP | Q08380 | up |  |  |
| VIME | P08670 | up |  |  |
| ZA2G | P25311 | up |  |  |
| PGS2 # | P07585 | up | high expression of these proteins are associated with lymph node metastasis | (139) |
| ENPL # | P14625 | up |  |  |
| PDIA3 # | P30101 | up | biomarker candidates for breast cancer tissues | (135) |
| DDX5 | P17844 | up | DDX5 is Up-regulated in Invasive Human Breast Cancers | (140) |
| LRC59 * | Q96AG4 | up | correlation with metastatic capability | (141) |
| CD59 * | P13987 | down |  |  |
| CSPG4 * | Q6UVK1 | down |  |  |
| ATPA * | P25705 | up | up-regulation of α-subunit of ATP synthase was identified in high metastatic cells compared with low metastatic cells | (142) |
| ANAX1 # | P04083 | up | significantly up-regulated in the model group and down-regulated upon treatment with RuXian-I | (143) |

**for TNBS protein biomarkers*

*#for protein markers obtained from studies conducted and validated in primary tumor tissues of BC patients*

*§ for Cancer Steam Cells (CSCs) protein biomarkers*

**Table S5: Biofluids Breast Cancer Metabolomics Biomarker**

| **Biomarkers** | **Kegg Code** | **Trend** | **Localization** | | **Analytical technique** | **Ref.** |
| --- | --- | --- | --- | --- | --- | --- |
| Phosphocholines |  | up/down | plasma | | LC-MS/MS | (144) |
| LysoPhosphocholines |  | down | plasma | | LC-MS/MS | (144) |
| Sphingomyelins |  | up | plasma | | LC-MS/MS | (144) |
| O-butanoylcarnitine | C02862 | up | plasma | | LC-MS/MS | (144) |
| Homovanillate | C05582 | down | urine/urine/  blood/  cerebrospinal fluid | | GC-MS, GC-MS,  GC-MS | (145, 146) |
| 4-hydroxyphenylacetate | C00642 | down/  down | urine/urine/  urine | | GC-MS, NMR,  GC-MS | (145-147) |
| 5-hydroxyindoleacetate | C05635 | down | urine/blood/  cerebrospinal fluid | | GC-MS, GC-MS | (145, 146) |
| Urea | C00086 | up/down | urine/urine/  blood/ cerebrospinal fluid | | GC-MS, GC-MS, NMR | (146, 147) |
| 5-hydroxymethyl-2-deoxyuridine | not available |  |  | | GC-MS | (148) |
| 8-hydroxy-2-deoxyguanosine | not available |  |  | | LC-MS | (148) |
| Histidine | C00135 | up/  down/up | serum/serum MBC/saliva | | NMR, GC-MS, NMR, GC-MS, HILIC and RPLC UPLC-ESI-MS | (149-151) |
| Proline | C00148 | down/up/up/up/up | serum/serum MBC/plasma/  serum/dried blood spot | | NMR, GC-MS, NMR, LC-MS/MS, GC-MS, NMR, GC-MS/MS | (149, 152-154) |
| N-acetylglycine | not available | down | serum | | NMR, GC-MS | (149) |
| 3-hydroxy-2-methyl butanoic acid | not available | down | serum | | NMR, GC-MS | (149) |
| Alanine | C00041 | down/  down/  down/  down/up | serum MBC/  urine/urine/  serum/plasma/  plasma | | NMR, NMR, GC-MS, LC-MS/MS, LC-TOFMS, GC-TOFMS, LC-MS/MS, GC-MS | (147, 150, 152, 155, 156) |
| Acetoacetate | C00164 | up | serum MBC | | NMR | (150) |
| 3-hydroxybutyrate | C01188 | up | serum MBC | | NMR | (150) |
| Glycerol | C00116 | up | serum MBC | | NMR | (150) |
| Phenylalanine | C00079 | up/up/up | serum MBC/  urine/saliva | | NMR, LC-MS/MS, HILIC and RPLC UPLC-ESI-MS | (150, 151, 153, 157) |
| Mannose | C00159 | up | serum MBC | | NMR | (150) |
| L-Lysine | C00047 | up/up/  down | serum MBC/plasma/  plasma | | NMR, NMR, LC-MS | (153, 158, 159) |
| N-acetylcysteine | C06809 | up | serum MBC | | NMR | (153) |
| Total free fatty acids |  | up | serum | | GC-MS | (160) |
| Palmitic acid | C00249 | up | serum | | GC-MS | (160) |
| Stearic acid | C01530 | up | serum | | GC-MS | (160) |
| Linoleic acid | C01595 | up | serum | | GC-MS | (160) |
| Creatine | C00300 | down/  down/up | urine/plasma/  plasma | | NMR, LC-MS, NMR | (147, 158, 159) |
| Acetate | C00033 | down | urine | | NMR | (147) |
| Succinate | C00042 | down/  down | urine/ serum, plasma | | NMR, LC-TOFMS, GC-TOFMS | (147, 156) |
| Levoglucosan | not available | down | urine | | NMR | (147) |
| Lactate | C00186 | down/up | urine/serum | | NMR, NMR, GC-MS | (147, 149) |
| Pyroglutamate | C01879 | down | urine | | NMR | (147) |
| Formate | C00058 | down/up | urine/serum | | NMR, NMR, GC-MS | (147, 149) |
| Isoleucine | C00407 | down | urine | | NMR | (147) |
| Sucrose | C00089 | down | urine | | NMR, LC-MS, GC-MS | (147) |
| Trigonelline | C01004 | down | urine | | NMR | (147) |
| Leucine | C00123 | down | urine | | NMR | (147) |
| Asparagine | C00152 | down/up | urine/dried blood spot | NMR, MS | | (147, 154) |
| Glucose | C00031 | down/up/up | urine/plasma/  serum MBC | | NMR | (147, 153, 159) |
| Ethanolamine | C00189 | down | urine | | NMR | (147) |
| Dimethylamine | C00543 | down | urine | | NMR | (147) |
| 4-hydroxyphenylacetate | C00642 | down | urine/urine | | NMR, GC-MS | (146, 147) |
| Creatinine | C00791 | down/up | urine/plasma | | NMR, NMR | (146, 147) |
| Glycerol-3-phosphate | C03189 | down | serum/plasma/  urine | | LC-TOFMS, GC-TOFMS, LC-MS/GC-MS | (155, 156) |
| Glycine | C00037 | down/  down | serum/plasma/  urine | | LC-TOFMS, GC-TOFMS, LC-MS, GC-MS | (155, 156) |
| Serine | C00065 | down | serum/plasma | | LC-TOFMS,  GC-TOFMS | (156) |
| Choline | C00114 | down/up | serum/plasma | | LC-TOFMS,  GC-TOFMS, NMR, GC-MS | (147) |
| Hippurate | C01586 | down | urine | | NMR | (147) |
| 1-methylnicotinamide | C02918 | down | urine | | NMR | (147) |
| Uracil | C00106 | down | urine | | NMR, LC-MS, GC-MS | (147, 155) |
| Valine | C00183 | down/up/  up/down | urine/plasma/  plasma/plasma | | NMR, LC-MS,  GC-MS, NMR, LC-MS | (146, 147, 152, 158) |
| *trans*-Aconitate | C02341 | down | urine | | NMR | (147) |
| Threonine | [C00188](https://www.genome.jp/dbget-bin/www_bget?cpd:C00188) | down | urine | | LC-MS, GC-MS | (155) |
| Alanine | C00041 | down | urine | | LC-MS, GC-MS | (155) |
| Glutamic acid | [C00025](https://www.genome.jp/dbget-bin/www_bget?cpd:C00025) | down | urine | | LC-MS, GC-MS | (155) |
| Cysteine | [C00097](https://www.genome.jp/dbget-bin/www_bget?cpd:C00097) | down | urine | | LC-MS, GC-MS | (155) |
| Tryptophan | [C00078](https://www.genome.jp/dbget-bin/www_bget?cpd:C00078) | down | urine | | LC-MS, GC-MS | (155) |
| Glycolic acid | [C00160](https://www.genome.jp/dbget-bin/www_bget?cpd:C00160) | down | urine | | LC-MS, GC-MS | (155) |
| Malonic acid | [C00383](https://www.genome.jp/dbget-bin/www_bget?cpd:C00383) | down | urine | | LC-MS, GC-MS | (155) |
| Maleic acid | [C00922](https://www.genome.jp/dbget-bin/www_bget?cpd:C00922) | down | urine | | LC-MS, GC-MS | (155) |
| Succinic acid | [C00036](https://www.genome.jp/dbget-bin/www_bget?cpd:C00036) | down | urine | | LC-MS, GC-MS | (155) |
| Aminomalonic acid | [C00872](https://www.genome.jp/dbget-bin/www_bget?cpd:C00872) | down | urine | | LC-MS, GC-MS | (155) |
| N-methyl-glutamic acid | [C01046](https://www.genome.jp/dbget-bin/www_bget?cpd:C01046) | down | urine | | LC-MS, GC-MS | (155) |
| N-acetylglutamic acid | [C00624](https://www.genome.jp/dbget-bin/www_bget?cpd:C00624) | down | urine | | LC-MS, GC-MS | (155) |
| Homovanillic acid | [C05582](https://www.genome.jp/dbget-bin/www_bget?cpd:C05582) | down | urine | | LC-MS, GC-MS | (155) |
| 4-hydroxymandelic acid | [C11527](https://www.genome.jp/dbget-bin/www_bget?cpd:C11527) | down | urine | | LC-MS, GC-MS | (155) |
| Citric acid | [C00158](https://www.genome.jp/dbget-bin/www_bget?cpd:C00158) | down | urine | | LC-MS, GC-MS | (155) |
| Adenine | [C21844](https://www.genome.jp/dbget-bin/www_bget?cpd:C21844) | down | urine | | LC-MS, GC-MS | (155) |
| Gulonic acid | [C00800](https://www.genome.jp/dbget-bin/www_bget?cpd:C00800) | down | urine | | LC-MS, GC-MS | (155) |
| Indoleacetic acid | C00954 | down | urine | | LC-MS, GC-MS | (155) |
| 5′-methylthioadenosine | [C00170](https://www.genome.jp/dbget-bin/www_bget?cpd:C00170) | down | urine | | LC-MS, GC-MS | (155) |
| Turanose | [C19636](https://www.genome.jp/dbget-bin/www_bget?cpd:C19636) | down | urine | | LC-MS, GC-MS | (155) |
| Decenoylcarnitine | [C03299](https://www.genome.jp/dbget-bin/www_bget?cpd:C03299) | down | urine | | LC-MS, GC-MS | (155) |
| Decatrienoylcarnitine | not available | down | urine | | LC-MS, GC-MS | (155) |
| Octenoylcarnitine | [C02838](https://www.genome.jp/dbget-bin/www_bget?cpd:C02838) | down | urine | | LC-MS, GC-MS | (155) |
| Cadaverine | [C01672](https://www.genome.jp/dbget-bin/www_bget?cpd:C01672) | up | saliva | | UPLC-ESI-MS/MS | (161) |
| Spermine | C00750 | up | saliva | | UPLC-ESI-MS/MS | (161) |
| N^1^-Acetylspermine | C02567 | up | saliva | | UPLC-ESI-MS/MS | (161) |
| Spermidine | C00315 | up | saliva | | UPLC-ESI-MS/MS | (161) |
| N^1^-Acetylspermidine | C00612 | up/up | saliva/saliva | | UPLC-ESI-MS/MS | (161, 162) |
| N^8^-Acetylspermidine | C01029 | up/up | saliva/saliva | | UPLC-ESI-MS/MS | (161, 162) |
| Citrulline | C00327 | up | saliva | | HILIC and RPLC, UPLC-ESI-MS | (151) |
| Phosphatidylserine (14:1) | C02737 | up/down | saliva/urine | | HILIC and RPLC UPLC-ESI-MS, LC-MS, GC-MS | (151, 155) |
| Phosphatidylserine (16:1) | C02737 | up | saliva | | HILIC and RPLC, UPLC-ESI-MS | (151) |
| Monoacylglycerol (0:0) | C01885 | up | saliva | | HILIC and RPLC, UPLC-ESI-MS | (151) |
| Monoacylglycerol (14:0) | C01885 | up | saliva | | HILIC and RPLC UPLC-ESI-MS | (151) |
| Lysophosphatidylethanolamine (18:2) | C04438 | up/down | saliva/  urine | | HILIC and RPLC, UPLC-ESI-MS, LC-MS, GC-MS | (151, 155) |
| Lysophosphatidylethanolamine (0:0) | C04438 | up | saliva | | HILIC and RPLC, UPLC-ESI-MS | (151) |
| Phosphatidylethanolamine (22:0) | C00350 | up | saliva | | HILIC and RPLC, UPLC-ESI-MS | (151) |
| Phosphatidylethanolamine (20:4) | C00350 | up | saliva | | HILIC and RPLC, UPLC-ESI-MS | (151) |
| N-Acetylneuraminic acid | C00270 | up | saliva | | HILIC and RPLC, UPLC-ESI-MS | (151) |
| 4-hydroxyphenylpyruvic acid | C01179 | up | saliva | | HILIC and RPLC, UPLC-ESI-MS | (151) |
| Total cholesterol |  | down | plasma | | Spectrophotometry | (163) |
| Dimethylarginine | C03626 | up | urine | | LC-MS/MS | (157) |
| Tyrosine | C00082 | up/up | urine/serum | LC-MS/MS,  NMR, GC-MS | | (149, 157) |
| Tyrosine | C00082 | up | urine | | LC-MS/MS | (157) |
| Pantothenic acid | C00864 | up | urine | | LC-MS/MS | (157) |
| Succinyladenosine | not available | up | urine | | LC-MS/MS | (157) |
| Threonylcarbamoyladenosin |  | up | urine | | LC-MS/MS | (157) |
| Tryptophan | C00078 | up | urine | | LC-MS/MS | (157) |
| Kynurenic acid | C01717 | up | urine | | LC-MS/MS | (157) |
| Nicotinuric acid | C05380 | up | urine | | LC-MS/MS | (157) |
| Indolelactic acid | C02043 | up | urine | | LC-MS/MS | (157) |
| 3-methyl-pentanoic acid | [C00077](https://www.genome.jp/dbget-bin/www_bget?cpd:C00077) | up | saliva | | GC-MS | (164) |
| p-tert-butyl-phenol | [C00146](https://www.genome.jp/dbget-bin/www_bget?cpd:C00146) | up | saliva | | GC-MS | (164) |
| Phenol | [C00146](https://www.genome.jp/dbget-bin/www_bget?cpd:C00146) | up | saliva | | GC-MS | (164) |
| Acetic acid | [C00033](https://www.genome.jp/dbget-bin/www_bget?cpd:C00033) | up | saliva | | GC-MS | (164) |
| Propanoic acid | [C00163](https://www.genome.jp/dbget-bin/www_bget?cpd:C00163) | up | saliva | | GC-MS | (164) |
| Benzoic acid | [C00180](https://www.genome.jp/dbget-bin/www_bget?cpd:C00180) | up | saliva | | GC-MS | (164) |
| 1,2-decanediol, 2-decanone | [C10482](https://www.genome.jp/dbget-bin/www_bget?cpd:C10482) | up | saliva | | GC-MS | (164) |
| Decanal | [C12307](https://www.genome.jp/dbget-bin/www_bget?cpd:C12307) | up | saliva | | GC-MS | (164) |
| Hypotaurine | C00519 | up | serum/plasma | | LC-TOFMS,  GC-TOFMS | (156) |
| Pyruvate | C00022 | up/up | serum/plasma/  serum MBC | | LC-TOFMS,  GC-TOFMS, NMR | (150, 156) |
| Carnitine | C00318 | up | plasma | | LC-MS, GC-MS | (152) |
| Lysophosphatidylcholine (20:4) | C04230 | up/down | plasma/urine | | LC-MS, GC-MS | (152, 155) |
| Lysophosphatidylcholine (16:1) | C04230 | up | plasma | | LC-MS, GC-MS | (152) |
| 2-octenedioic acid | not available | up/down | plasma/plasma | | LC-MS, GC-MS,  LC-MS | (152, 158) |
| 2-hydroxy-3-methylbutyric acid | [C00141](https://www.genome.jp/dbget-bin/www_bget?cpd:C00141) | up | plasma | | LC-MS | (158) |
| 2-hydroxy-3- methylpentanoic acid | [C02486](https://www.genome.jp/dbget-bin/www_bget?cpd:C02486) | up | plasma | | LC-MS | (158) |
| 3-methylglutaric acid | [C03761](https://www.genome.jp/dbget-bin/www_bget?cpd:C03761) | up | plasma | | LC-MS | (158) |
| 7-ketocholesterol | not available | up | plasma | | LC-MS | (158) |
| Chenodeoxycholic acid | [C02528](https://www.genome.jp/dbget-bin/www_bget?cpd:C02528) | up | plasma | | LC-MS | (158) |
| Cytidine | [C00055](https://www.genome.jp/dbget-bin/www_bget?cpd:C00055) | up | plasma | | LC-MS | (158) |
| DL-pipecolic acid | [C00408](https://www.genome.jp/dbget-bin/www_bget?cpd:C00408) | up | plasma | | LC-MS | (158) |
| Dopamine | [C03758](https://www.genome.jp/dbget-bin/www_bget?cpd:C03758) | up | plasma | | LC-MS | (158) |
| Homocystine | [C01817](https://www.genome.jp/dbget-bin/www_bget?cpd:C01817) | up | plasma | | LC-MS | (158) |
| Inosine diphosphate (IDP) | [C00104](https://www.genome.jp/dbget-bin/www_bget?cpd:C00104) | up | plasma | | LC-MS | (158) |
| Linoleic acid | [C01595](https://www.genome.jp/dbget-bin/www_bget?cpd:C01595) | up | plasma | | LC-MS | (158) |
| Myristic acid | [C06424](https://www.genome.jp/dbget-bin/www_bget?cpd:C06424) | up | plasma | | LC-MS | (158) |
| Oleamide | [C19670](https://www.genome.jp/dbget-bin/www_bget?cpd:C19670) | up | plasma | | LC-MS | (158) |
| Stearic acid | [C01530](https://www.genome.jp/dbget-bin/www_bget?cpd:C01530) | up | plasma | | LC-MS | (158) |
| Taurine | [C00245](https://www.genome.jp/dbget-bin/www_bget?cpd:C00245) | up | plasma | | LC-MS | (158) |
| Threonate | [C01620](https://www.genome.jp/dbget-bin/www_bget?cpd:C01620) | up | plasma | | LC-MS | (158) |
| Uric acid | [C00366](https://www.genome.jp/dbget-bin/www_bget?cpd:C00366) | up | plasma | | LC-MS | (158) |
| 2-methylhippuric acid | C01586 | down | plasma | | LC-MS | (158) |
| 3-hydroxyanthranilic acid | C00632 | down | plasma | | LC-MS | (158) |
| 4-acetamidobutanoate | C02946 | down | plasma | | LC-MS | (158) |
| 5β-cholestane-3α,7α,12α-triol | C05454 | down | plasma | | LC-MS | (158) |
| 5α-androstane-3,17-dione | C00674 | down | plasma | | LC-MS | (158) |
| 7α-hydroxy-cholesterol | C03594 | down | plasma | | LC-MS | (158) |
| Caproic acid | C01585 | down | plasma | | LC-MS | (158) |
| Cortisol | C00735 | down | plasma | | LC-MS | (158) |
| Cortisone | C00762 | down | plasma | | LC-MS | (158) |
| Hippuric acid | C01586 | down/  down | plasma/urine | | LC-MS, GC-MS | (155, 158) |
| L-Arginine | C00062 | down/up | plasma/plasma | | LC-MS, NMR | (148, 158) |
| Retinoic acid | C00777 | down | plasma | | LC-MS | (158)\ |
| Glutamine | C00303 | down/up | plasma/plasma | | LC-MS, NMR | (148, 158) |
| Ceramide (18:1) | C00195 | down | plasma | | LC-MS | (158) |
| 3-hydroxybutyrate | C01089 | up | serum | NMR, GC-MS | | (149) |
| Glutamate | C00025 | up/up | serum/serum MBC | NMR, GC-MS, NMR | | (149, 150) |
| Nonanedioic Acid | C08261 | up | serum | NMR, GC-MS | | (149) |
| N-Acylglycine | C02055 | up | serum | NMR, GC-MS | | (149) |
| Lysophosphatidylcholine (18:2) | C04230 | down | saliva | HILIC and RPLC, UPLC-ESI-MS | | (151) |
| Lysophosphatidylcholine (18:1) | C04230 | up | saliva | HILIC and RPLC, UPLC-ESI-MS | | (151) |
| Lysophosphatidylcholine (16:0) | C04230 | up | saliva | HILIC and RPLC, UPLC-ESI-MS | | (151) |
| Lysophosphatidylcholine (22:6) | C04230 | up | saliva | HILIC and RPLC, UPLC-ESI-MS | | (151) |
| Palmitic amide | not available | down | saliva | HILIC and RPLC, UPLC-ESI-MS | | (151) |
| Phytosphingosine | C12144 | down | saliva | HILIC and RPLC, UPLC-ESI-MS | | (151) |
| N-Acetyl-L-Phenylalanine | C03519 | down | saliva | HILIC and RPLC, UPLC-ESI-MS | | (151) |
| Propionylcholine | not available | down | saliva | HILIC and RPLC, UPLC-ESI-MS | | (151) |
| N-acetyl-putrescine | C02714 | up | saliva | UPLC-ESI-MS/MS | | (162) |
| 3-(Cystein-S-Yl)Acetaminophen | not available | up | plasma | UHPLC-MS,  GC-MS | | (165) |
| Cysteine S-Sulfate | C05824 | up | plasma | UHPLC-MS,  GC-MS | | (165) |
| Indoleacetylglutamine | not available | down | plasma | UHPLC-MS,  GC-MS | | (165) |
| Sphingosine | C00319 | down | plasma | UHPLC-MS,  GC-MS | | (165) |
| Adenine | C00147 | down | plasma | LC-MS/MS | | (166) |
| N^6^-Methyladenosine | not available | down | plasma | LC-MS/MS | | (166) |
| 1-methylguanine | C04152 | down | plasma | LC-MS/MS | | (166) |

**Table 6: Cell/Tissue Breast Cancer Metabolomics Biomarkers**

| **Biomarkers** | **Kegg Code** | **Trend** | **Localization** | **Analytical technique** | **Ref.** |
| --- | --- | --- | --- | --- | --- |
| Glycine | C00037 | up/up/up/  up/up | tissue/tissue/  tissue/tissue/  tissue | HR-MAS, NMR, HR-MAS, NMR,  HR-MAS, NMR,  LC-MS, GC-MS | (167-172) |
| Lactate | C00186 | up/up/up/  up/up | tissue/tissue/  tissue/tissue/  tissue | HR-MAS, NMR, HR-MAS, NMR, LC-ESI-MS, HR-MAS, NMR | (168-173) |
| Choline | C00114 | up/up/up/  up/up | tissue/tissue/  tissue/tissue/  tissue | HR-MAS, NMR, LC-ESI-MS, HR-MAS, NMR, HR-MAS, NMR, LC-MS, GC-MS | (167, 169-171, 173, 174) |
| Glycerol-3-phosphate | C03189 | up | tissue |  | (175, 176) |
| Glycerophosphocholine | C00670 | down/up/up | tissue/tissue/  tissue | HR-MAS, NMR, LC-ESI-MS | (169, 173, 177) |
| Phosphocholine | C00588 | up/up/up/  up/up/up/up | tissue/tissue/  tissue/tissue/  tissue/tissue/  tissue | HPLC-ESI-MS,  LC-MS, NMR, HR-MAS, NMR, HR-MAS, NMR, LC-ESI-MS,  HR-MAS, NMR | (169, 171, 173, 176-180) |
| Phosphatidylethanolamines | C00350 | up/up/up | tissue/breast cancer cell line/tissue | LC-MS/MS,  GC-MS, LC-MS/MS | (176, 178, 181) |
| Phosphatidylcholine | C00157 | up/up | breast cancer cell line/tissue | LC-MS/MS,  GC-MS, LC-MS | (176, 181) |
| Lysophosphatidylcholine | C04230 | up | breast cancer cell line | LC-MS/MS | (181) |
| Total Choline |  | up | tissue | LC-MS, NMR | (179) |
| Glycerol | C00116 | down | tissue | GC-TOF-MS | (182) |
| Glucuronic acid | C00191 | down | tissue | GC-TOF-MS | (182) |
| N-methylalanine | C02721 | down | tissue | GC-TOF-MS | (182) |
| Cytidine-5 monophosphate | C00055 | up | tissue | GC-TOF-MS | (183) |
| Adenosine-5 monophosphate | C00020 | up | tissue | GC-TOF-MS | (183) |
| Phosphoethanolamine | C00346 | up/up | tissue/tissue | GC-TOF-MS, NMR | (180, 183) |
| Taurine | C00245 | up/up/up/  up/up/up | tissue/tissue/  tissue/tissue/  tissue/tissue | GC-TOF-MS, HR-MAS, NMR,  HR-MAS, NMR, LC-ESI-MS, HR-MAS, NMR | (169, 171-173, 180, 183) |
| Pyrazine 2,5-dihydroxy | C20515 | up | tissue | GC-TOF-MS | (183) |
| Creatinine | C00791 | up | tissue | GC-TOF-MS | (183) |
| Hypoxanthine | C00262 | up | tissue | GC-TOF-MS | (183) |
| Glycerol-3-phosphate | C03189 | up | tissue | GC-TOF-MS | (183) |
| Aminomalonate | C00872 | up | tissue | GC-TOF-MS | (183) |
| Glutamic acid | C00025 | up/up | tissue/tissue | GC-TOF-MS | (182, 183) |
| Malate | C00149 | up/up | tissue/tissue | GC-TOF-MS | (182, 183) |
| Oxoproline | C01877 | up | tissue | GC-TOF-MS | (183) |
| Inosine | C00294 | down | breast cancer cell line | HPLC-QqQ-MS | (184) |
| Adenosine | C00212 | down | breast cancer cell line | HPLC-QqQ-MS | (184) |
| 2-pentanone | C01949 | up | breast cancer cell line | GC-MS | (185) |
| 2-heptanone | C08380 | up | breast cancer cell line | GC-MS | (185) |
| Ethyl acetate | C00849 | up | breast cancer cell line | GC-MS | (185) |
| Ethyl propanoate | C00163 | up | breast cancer cell line | GC-MS | (185) |
| 2-methyl butanoate | C00141 | up | breast cancer cell line | GC-MS | (185) |
| Uridine | C00299 | down/up | breast cancer cell line/tissue | HPLC-QqQ-MS,  LC-MS, GC-MS | (170, 184) |
| Ascorbate | C00072 | up | tissue | HR-MAS, NMR | (169) |
| Creatine | C00300 | up/up | tissue/tissue | HR-MAS, NMR, HR-MAS MR | (169, 171) |
| Phosphatidylinositol | C04195 | up | tissue | GC-MS, LC-MS | (176) |
| Sphingomyelin | C00550 | up | tissue | GC-MS, LC-MS | (176) |
| Triglycerides | C00422 | up | tissue | GC-MS, LC-MS | (176) |
| N-acetylalanine | [C01073](https://www.genome.jp/dbget-bin/www_bget?cpd:C01073) | up | tissue | LC-MS, GC-MS | (170) |
| N-acetylaspartate | [C01042](https://www.genome.jp/dbget-bin/www_bget?cpd:C01042) | up | tissue | LC-MS, GC-MS | (170) |
| Cystathionine | [C00542](https://www.genome.jp/dbget-bin/www_bget?cpd:C00542) | up | tissue | LC-MS, GC-MS | (170) |
| N-acetylmethionine | [C02712](https://www.genome.jp/dbget-bin/www_bget?cpd:C02712) | up | tissue | LC-MS, GC-MS | (170) |
| S-adenosylhomocysteine | [C00021](https://www.genome.jp/dbget-bin/www_bget?cpd:C00021) | up | tissue | LC-MS, GC-MS | (170) |
| Glutamate | [C00025](https://www.genome.jp/dbget-bin/www_bget?cpd:C00025) | up | tissue | LC-MS, GC-MS | (170) |
| 5-oxoproline | [C01879](https://www.genome.jp/dbget-bin/www_bget?cpd:C01879) | up | tissue | LC-MS, GC-MS | (170) |
| N-acetylglucosamine | [C00043](https://www.genome.jp/dbget-bin/www_bget?cpd:C00043) | up | tissue | LC-MS, GC-MS | (170) |
| Betaine | [C00719](https://www.genome.jp/dbget-bin/www_bget?cpd:C00719) | up | tissue | LC-MS, GC-MS | (170) |
| N-acetylthreonine | not available | up | tissue | LC-MS, GC-MS | (170) |
| Threonine | [C00188](https://www.genome.jp/dbget-bin/www_bget?cpd:C00188) | up | tissue | LC-MS, GC-MS | (170) |
| 3-(4-hydroxyphenyl)lactate | [C03964](https://www.genome.jp/dbget-bin/www_bget?cpd:C03964) | up | tissue | LC-MS, GC-MS | (170) |
| Putrescine | [C00134](https://www.genome.jp/dbget-bin/www_bget?cpd:C00134) | up | tissue | LC-MS, GC-MS | (170) |
| Aspartate | [C00049](https://www.genome.jp/dbget-bin/www_bget?cpd:C00049) | up | tissue | LC-MS, GC-MS | (170) |
| Tryptophan | [C00078](https://www.genome.jp/dbget-bin/www_bget?cpd:C00078) | up | tissue | LC-MS, GC-MS | (170) |
| Dimethylarginine | [C03626](https://www.genome.jp/dbget-bin/www_bget?cpd:C03626) | up | tissue | LC-MS, GC-MS | (170) |
| Proline | [C00148](https://www.genome.jp/dbget-bin/www_bget?cpd:C00148) | up | tissue | LC-MS, GC-MS | (170) |
| Stachydrine | [C10172](https://www.genome.jp/dbget-bin/www_bget?cpd:C10172) | up | tissue | LC-MS, GC-MS | (170) |
| *trans*-4-hydroxyproline | [C05147](https://www.genome.jp/dbget-bin/www_bget?cpd:C05147) | up | tissue | LC-MS, GC-MS | (170) |
| 2-methylbutyroylcarnitine | [C02301](https://www.genome.jp/dbget-bin/www_bget?cpd:C02301) | up | tissue | LC-MS, GC-MS | (170) |
| Urea | [C00086](https://www.genome.jp/dbget-bin/www_bget?cpd:C00086) | up | tissue | LC-MS, GC-MS | (170) |
| Erythronate | [C03064](https://www.genome.jp/dbget-bin/www_bget?cpd:C03064) | up | tissue | LC-MS, GC-MS | (170) |
| N-acetylglucosamine | [C00043](https://www.genome.jp/dbget-bin/www_bget?cpd:C00043) | up | tissue | LC-MS, GC-MS | (170) |
| Fucose | [C02095](https://www.genome.jp/dbget-bin/www_bget?cpd:C02095) | up | tissue | LC-MS, GC-MS | (170) |
| N-acetylglucosamine | [C00043](https://www.genome.jp/dbget-bin/www_bget?cpd:C00043) | up | tissue | LC-MS, GC-MS | (170) |
| 6-phosphate N-acetylneuraminate | [C06241](https://www.genome.jp/dbget-bin/www_bget?cpd:C06241) | up | tissue | LC-MS, GC-MS | (170) |
| Fructose | [C10906](https://www.genome.jp/dbget-bin/www_bget?cpd:C10906) | up | tissue | LC-MS, GC-MS | (170) |
| Maltose | [C01971](https://www.genome.jp/dbget-bin/www_bget?cpd:C01971) | up | tissue | LC-MS, GC-MS | (170) |
| Maltotetraose | [C02052](https://www.genome.jp/dbget-bin/www_bget?cpd:C02052) | up | tissue | LC-MS, GC-MS | (170) |
| maltotriose | [C01835](https://www.genome.jp/dbget-bin/www_bget?cpd:C01835) | up | tissue | LC-MS, GC-MS | (170) |
| Mannose-6-phosphate | [C00275](https://www.genome.jp/dbget-bin/www_bget?cpd:C00275) | up | tissue | LC-MS, GC-MS | (170) |
| 3-phosphoglycerate | [C00197](https://www.genome.jp/dbget-bin/www_bget?cpd:C00197) | up | tissue | LC-MS, GC-MS | (170) |
| 1-palmitoylplasmenylethanolamine | [C04756](https://www.genome.jp/dbget-bin/www_bget?cpd:C04756) | up | tissue | LC-MS, GC-MS | (170) |
| Fructose-6-phosphate | [C00085](https://www.genome.jp/dbget-bin/www_bget?cpd:C00085) | up | tissue | LC-MS, GC-MS | (170) |
| Glucose-6-phosphate (G6P) | [C00092](https://www.genome.jp/dbget-bin/www_bget?cpd:C00092) | up | tissue | LC-MS, GC-MS | (170) |
| Lactate | [C00186](https://www.genome.jp/dbget-bin/www_bget?cpd:C00186) | up | tissue | LC-MS, GC-MS | (170) |
| Phosphoenolpyruvate (PEP) | [C00074](https://www.genome.jp/dbget-bin/www_bget?cpd:C00074) | up | tissue | LC-MS, GC-MS | (170) |
| Gluconate | [C00257](https://www.genome.jp/dbget-bin/www_bget?cpd:C00257) | up | tissue | LC-MS, GC-MS | (170) |
| Ribulose 5-phosphate | [C00199](https://www.genome.jp/dbget-bin/www_bget?cpd:C00199) | up | tissue | LC-MS, GC-MS | (170) |
| Xylulose 5-phosphate | [C00231](https://www.genome.jp/dbget-bin/www_bget?cpd:C00231) | up | tissue | LC-MS, GC-MS | (170) |
| Ribose | [C00121](https://www.genome.jp/dbget-bin/www_bget?cpd:C00121) | up | tissue | LC-MS, GC-MS | (170) |
| Ribulose | [C05052](https://www.genome.jp/dbget-bin/www_bget?cpd:C05052) | up | tissue | LC-MS, GC-MS | (170) |
| Ascorbate | [C00072](https://www.genome.jp/dbget-bin/www_bget?cpd:C00072) | up | tissue | LC-MS, GC-MS | (170) |
| Threonate | [C01620](https://www.genome.jp/dbget-bin/www_bget?cpd:C01620) | up | tissue | LC-MS, GC-MS | (170) |
| 1-methylnicotinamide | [C02918](https://www.genome.jp/dbget-bin/www_bget?cpd:C02918) | up | tissue | LC-MS, GC-MS | (170) |
| Pantothenate phosphate | [C03492](https://www.genome.jp/dbget-bin/www_bget?cpd:C03492) | up | tissue | LC-MS, GC-MS | (170) |
| Pyrophosphate | [C00013](https://www.genome.jp/dbget-bin/www_bget?cpd:C00013) | up | tissue | LC-MS, GC-MS | (170) |
| 3-dehydrocarnitine | [C02636](https://www.genome.jp/dbget-bin/www_bget?cpd:C02636) | up | tissue | LC-MS, GC-MS | (170) |
| Acetylcarnitine | [C02571](https://www.genome.jp/dbget-bin/www_bget?cpd:C02571) | up | tissue | LC-MS, GC-MS | (170) |
| Dihomo-linolenate | [C06427](https://www.genome.jp/dbget-bin/www_bget?cpd:C06427) | up | tissue | LC-MS, GC-MS | (170) |
| Docosahexaenoate | [C06429](https://www.genome.jp/dbget-bin/www_bget?cpd:C06429) | up | tissue | LC-MS, GC-MS | (170) |
| Eicosapentaenoate | [C06428](https://www.genome.jp/dbget-bin/www_bget?cpd:C06428) | up | tissue | LC-MS, GC-MS | (170) |
| 2-hydroxypalmitate | [C18218](https://www.genome.jp/dbget-bin/www_bget?cpd:C18218) | up | tissue | LC-MS, GC-MS | (170) |
| 4-hydroxyphenylpyruvate | [C01179](https://www.genome.jp/dbget-bin/www_bget?cpd:C01179) | up | tissue | LC-MS, GC-MS | (170) |
| Cytidine | [C00475](https://www.genome.jp/dbget-bin/www_bget?cpd:C00475) | up | tissue | LC-MS, GC-MS | (170) |
| Cytidine-5'-diphosphocholine | [C00307](https://www.genome.jp/dbget-bin/www_bget?cpd:C00307) | up | tissue | LC-MS, GC-MS | (170) |
| Ethanolamine | [C00189](https://www.genome.jp/dbget-bin/www_bget?cpd:C00189) | up | tissue | LC-MS, GC-MS | (170) |
| Inositol 1-phosphate (I1P) | [C01177](https://www.genome.jp/dbget-bin/www_bget?cpd:C01177) | up | tissue | LC-MS, GC-MS | (170) |
| Adrenate | [C16527](https://www.genome.jp/dbget-bin/www_bget?cpd:C16527) | up | tissue | LC-MS, GC-MS | (170) |
| Arachidonate | [C00219](https://www.genome.jp/dbget-bin/www_bget?cpd:C00219) | up | tissue | LC-MS, GC-MS | (170) |
| *cis*-vaccenate | [C21944](https://www.genome.jp/dbget-bin/www_bget?cpd:C21944) | up | tissue | LC-MS, GC-MS | (170) |
| Oleate | [C03425](https://www.genome.jp/dbget-bin/www_bget?cpd:C03425) | up | tissue | LC-MS, GC-MS | (170) |
| Palmitate | [C00249](https://www.genome.jp/dbget-bin/www_bget?cpd:C00249) | up | tissue | LC-MS, GC-MS | (170) |
| Stearidonate | [C16300](https://www.genome.jp/dbget-bin/www_bget?cpd:C16300) | up | tissue | LC-MS, GC-MS | (170) |
| 1-oleoylglycerophosphocholine | [C03916](https://www.genome.jp/dbget-bin/www_bget?cpd:C03916) | up | tissue | LC-MS, GC-MS | (170) |
| 1-oleoylglycerophosphoethanolamine | [C04475](https://www.genome.jp/dbget-bin/www_bget?cpd:C04475) | up | tissue | LC-MS, GC-MS | (170) |
| 1-palmitoylglycerophosphocholine | [C04102](https://www.genome.jp/dbget-bin/www_bget?cpd:C04102) | up | tissue | LC-MS, GC-MS | (170) |
| 1-stearoylglycerophosphocholine | [C04230](https://www.genome.jp/dbget-bin/www_bget?cpd:C04230) | up | tissue | LC-MS, GC-MS | (170) |
| 1-stearoylglycerophosphoethanolamine | [C04475](https://www.genome.jp/dbget-bin/www_bget?cpd:C04475) | up | tissue | LC-MS, GC-MS | (170) |
| 1-stearoylglycerophosphoinositol | [C03819](https://www.genome.jp/dbget-bin/www_bget?cpd:C03819) | up | tissue | LC-MS, GC-MS | (170) |
| 2-arachidonoylglycerophosphocholine | [C04230](https://www.genome.jp/dbget-bin/www_bget?cpd:C04230) | up | tissue | LC-MS, GC-MS | (170) |
| 2-arachidonoylglycerophosphoethanolamine | [C04475](https://www.genome.jp/dbget-bin/www_bget?cpd:C04475) | up | tissue | LC-MS, GC-MS | (170) |
| 2-docosahexaenoylglycerophosphoethanolamine | [C04475](https://www.genome.jp/dbget-bin/www_bget?cpd:C04475) | up | tissue | LC-MS, GC-MS | (170) |
| 2-linoleoylglycerophosphoethanolamine | [C04475](https://www.genome.jp/dbget-bin/www_bget?cpd:C04475) | up | tissue | LC-MS, GC-MS | (170) |
| 2-oleoylglycerophosphocholine | [C03916](https://www.genome.jp/dbget-bin/www_bget?cpd:C03916) | up | tissue | LC-MS, GC-MS | (170) |
| 2-oleoylglycerophosphoethanolamine | [C04475](https://www.genome.jp/dbget-bin/www_bget?cpd:C04475) | up | tissue | LC-MS, GC-MS | (170) |
| 2-palmitoylglycerophosphocholine | [C04102](https://www.genome.jp/dbget-bin/www_bget?cpd:C04102) | up | tissue | LC-MS, GC-MS | (170) |
| Sphinganine | [C00836](https://www.genome.jp/dbget-bin/www_bget?cpd:C00836) | up | tissue | LC-MS, GC-MS | (170) |
| Sphingosine | [C00319](https://www.genome.jp/dbget-bin/www_bget?cpd:C00319) | up | tissue | LC-MS, GC-MS | (170) |
| Sphingomyelin | [C00550](https://www.genome.jp/dbget-bin/www_bget?cpd:C00550) | up | tissue | LC-MS, GC-MS | (170) |
| Hypoxanthine | [C00262](https://www.genome.jp/dbget-bin/www_bget?cpd:C00262) | up | tissue | LC-MS, GC-MS | (170) |
| Adenosine | [C00212](https://www.genome.jp/dbget-bin/www_bget?cpd:C00212) | up | tissue | LC-MS, GC-MS | (170) |
| N^1^-methyladenosine | [C02494](https://www.genome.jp/dbget-bin/www_bget?cpd:C02494) | up | tissue | LC-MS, GC-MS | (170) |
| Guanine | [C00242](https://www.genome.jp/dbget-bin/www_bget?cpd:C00242) | up | tissue | LC-MS, GC-MS | (170) |
| Guanosine | [C00387](https://www.genome.jp/dbget-bin/www_bget?cpd:C00387) | up | tissue | LC-MS, GC-MS | (170) |
| Cytidine 5'-monophosphate | [C00055](https://www.genome.jp/dbget-bin/www_bget?cpd:C00055) | up | tissue | LC-MS, GC-MS | (170) |
| Pseudouridine | [C02067](https://www.genome.jp/dbget-bin/www_bget?cpd:C02067) | up | tissue | LC-MS, GC-MS | (170) |
| Inositol | C00137 | up | tissue | HR-MAS MR | (171) |
| UDP-N-acetylgalactosamine | C00203 | up | tissue | NMR | (180) |
| 3-phosphoglycerate | C00597 | up | tissue | GC-TOF-MS | (182) |
| β-Alanine | C00099 | up | tissue | GC-TOF-MS | (182) |
| Fumaric Acid | C00122 | up | tissue | GC-TOF-MS | (182) |
| 2-Hydroxyglutaric Acid | C01087 | up | tissue | GC-TOF-MS | (182) |
| Serine | C00716 | up | tissue | GC-TOF-MS | (182) |
| Threonic Acid | C01620 | up | tissue | GC-TOF-MS | (182) |
| 2-Aminoadipic acid | [C00956](https://www.genome.jp/dbget-bin/www_bget?cpd:C00956) | up | tissue | GC-TOF-MS | (182) |
| Citrulline | C00327 | up | tissue | GC-TOF-MS | (182) |
| Glyceric Acid | C00258 | up | tissue | GC-TOF-MS | (182) |
| Uracil | C00106 | up | tissue | GC-TOF-MS,  LC-MS, GC-MS | (170, 182) |
| Myo-Inositol | C00137 | down | tissue | NMR | (170, 180) |
| UDP-N-Acetylglucosamine | C00043 | up | tissue | NMR | (180) |
| Glucose | C00031 | down | tissue | NMR | (180) |
| Ceramide (16:0) | C00195 | up | tissue | LC-MS/MS | (186) |
| Ceramide (24:1) | not available | up | tissue | LC-MS/MS | (186) |
| Ceramide (24:0) | not available | up | tissue | LC-MS/MS | (186) |

Data processing and elaboration

In order to combine the results from different “omics” studies, we introduced a new methodical framework by performing a meta-analysis through data “omics” integration.

In particular, we used Ingenuity Pathway Analysis software (IPA, Qiagen, Hilden, Germany) for “Core Analysis” to map statistically each gene or protein or metabolite for their functional annotation, such as network discovery, Upstream Regulator Analysis (URA) and downstream effects networks. Details of data processing and elaboration by IPA are fully described in Supplementary Materials. As reported in the previous tables, we have evaluated the single trends of each probable molecular biomarker (genes, proteins and metabolites) in the BC tissue and/or cells compared with controls. In this first scenario, we started from each single “omics” analysis, whose datasets were created in a separate data matrix for the three “omics” platforms (e.g. genomics, proteomics, and metabolomics). Dataset from genomics studies were uploaded on IPA software as a matrix where the rows in every data matrix correspond to a single gene with a measurement of its expression (0.01 for low, 0.1 for moderate-low, 10 for high-moderate, 100 for high Gene penetrance, as specified in Supplementary Table S1). At the same time, proteomics and metabolomics datasets were used individually for functional enrichment analyses on IPA by uploading their own expression (0.1 for down-regulation and 10 for up-regulation, see above Supplementary Tables S4 and S6).

At a later time, we performed a multi-omics integration strategy by simultaneously including and combining multiple datasets from different “omics” platforms. In particular, single data from each “omics” study were merged in a unique data matrix and uploaded on IPA software in order to build a cross-platform in which we better assessed new key pathways or functions related to BC.

According to IPA tool’s statistics, in all cases we considered molecules and/or relationships in all species and a confidence setting as high predicted or experimental observed (excluding medium predicted). IPA is able to identify relationships and pathways relevant to the uploaded dataset. In particular, it provides the principal diseases and function categories resulting from some of the modulated molecules of the uploaded dataset. Instead, URA is based on prior knowledge of expected effects and relationships between transcriptional regulators and their target genes from published literature citations stored in the IPA system (187). The p-value is a measurement of the statistical overlap between the protein dataset and the genes or function categories, and the significance is attributed to p-value < 0.05. Instead, the predicted activation or inhibition of each transcriptional regulator or downstream is inferred by the z-score generated by IPA system (z-scores ≥ 2.0 indicate that a molecule is activated, whereas z-scores ≤ -2.0 indicate the inhibition of target molecules). In fact, disease and functions and upstream regulators will be listed below with their own predicted z-score.

References

1. Chen S, Parmigiani G. Meta-analysis of BRCA1 and BRCA2 penetrance. Journal of clinical oncology : official journal of the American Society of Clinical Oncology. 2007;25(11):1329-33.

2. Rowan E, Poll A, Narod SA. A prospective study of breast cancer risk in relatives of BRCA1/BRCA2 mutation carriers. Journal of medical genetics. 2007;44(8):e89; author reply e8.

3. Somasundaram K. BRCA1 and BRCA1 Genes and Inherited Breast and/or Ovarian Cancer: Benefits of Genetic Testing. Indian journal of surgical oncology. 2010;1(3):245-9.

4. Garber JE, Goldstein AM, Kantor AF, Dreyfus MG, Fraumeni JF, Jr., Li FP. Follow-up study of twenty-four families with Li-Fraumeni syndrome. Cancer research. 1991;51(22):6094-7.

5. Harris CC, Hollstein M. Clinical implications of the p53 tumor-suppressor gene. The New England journal of medicine. 1993;329(18):1318-27.

6. Eng C. Genetics of Cowden syndrome: through the looking glass of oncology. International journal of oncology. 1998;12(3):701-10.

7. Freihoff D, Kempe A, Beste B, Wappenschmidt B, Kreyer E, Hayashi Y, et al. Exclusion of a major role for the PTEN tumour-suppressor gene in breast carcinomas. British journal of cancer. 1999;79(5-6):754-8.

8. Liaw D, Marsh DJ, Li J, Dahia PL, Wang SI, Zheng Z, et al. Germline mutations of the PTEN gene in Cowden disease, an inherited breast and thyroid cancer syndrome. Nature genetics. 1997;16(1):64-7.

9. Marsh DJ, Coulon V, Lunetta KL, Rocca-Serra P, Dahia PL, Zheng Z, et al. Mutation spectrum and genotype-phenotype analyses in Cowden disease and Bannayan-Zonana syndrome, two hamartoma syndromes with germline PTEN mutation. Human molecular genetics. 1998;7(3):507-15.

10. Starink TM, van der Veen JP, Arwert F, de Waal LP, de Lange GG, Gille JJ, et al. The Cowden syndrome: a clinical and genetic study in 21 patients. Clinical genetics. 1986;29(3):222-33.

11. Alenezi WM, Fierheller CT, Recio N, Tonin PN. Literature Review of BARD1 as a Cancer Predisposing Gene with a Focus on Breast and Ovarian Cancers. Genes. 2020;11(8).

12. Irminger-Finger I, Soriano JV, Vaudan G, Montesano R, Sappino AP. In vitro repression of Brca1-associated RING domain gene, Bard1, induces phenotypic changes in mammary epithelial cells. The Journal of cell biology. 1998;143(5):1329-39.

13. Meijers-Heijboer H, van den Ouweland A, Klijn J, Wasielewski M, de Snoo A, Oldenburg R, et al. Low-penetrance susceptibility to breast cancer due to CHEK2(*)1100delC in noncarriers of BRCA1 or BRCA2 mutations. Nature genetics. 2002;31(1):55-9.

14. Naidoo K, Wai PT, Maguire SL, Daley F, Haider S, Kriplani D, et al. Evaluation of CDK12 Protein Expression as a Potential Novel Biomarker for DNA Damage Response-Targeted Therapies in Breast Cancer. Molecular cancer therapeutics. 2018;17(1):306-15.

15. Rainville I, Hatcher S, Rosenthal E, Larson K, Bernhisel R, Meek S, et al. High risk of breast cancer in women with biallelic pathogenic variants in CHEK2. Breast cancer research and treatment. 2020;180(2):503-9.

16. Tien JF, Mazloomian A, Cheng SG, Hughes CS, Chow CCT, Canapi LT, et al. CDK12 regulates alternative last exon mRNA splicing and promotes breast cancer cell invasion. Nucleic acids research. 2017;45(11):6698-716.

17. Buys SS, Sandbach JF, Gammon A, Patel G, Kidd J, Brown KL, et al. A study of over 35,000 women with breast cancer tested with a 25-gene panel of hereditary cancer genes. Cancer. 2017;123(10):1721-30.

18. Couch FJ, Hart SN, Sharma P, Toland AE, Wang X, Miron P, et al. Inherited mutations in 17 breast cancer susceptibility genes among a large triple-negative breast cancer cohort unselected for family history of breast cancer. Journal of clinical oncology : official journal of the American Society of Clinical Oncology. 2015;33(4):304-11.

19. Vargas AC, Reis-Filho JS, Lakhani SR. Phenotype-genotype correlation in familial breast cancer. Journal of mammary gland biology and neoplasia. 2011;16(1):27-40.

20. Borresen AL, Andersen TI, Tretli S, Heiberg A, Moller P. Breast cancer and other cancers in Norwegian families with ataxia-telangiectasia. Genes, chromosomes & cancer. 1990;2(4):339-40.

21. Morrell D, Chase CL, Swift M. Cancers in 44 families with ataxia-telangiectasia. Cancer genetics and cytogenetics. 1990;50(1):119-23.

22. Pippard EC, Hall AJ, Barker DJ, Bridges BA. Cancer in homozygotes and heterozygotes of ataxia-telangiectasia and xeroderma pigmentosum in Britain. Cancer research. 1988;48(10):2929-32.

23. Swift M, Morrell D, Massey RB, Chase CL. Incidence of cancer in 161 families affected by ataxia-telangiectasia. The New England journal of medicine. 1991;325(26):1831-6.

24. Swift M, Reitnauer PJ, Morrell D, Chase CL. Breast and other cancers in families with ataxia-telangiectasia. The New England journal of medicine. 1987;316(21):1289-94.

25. Bernstein JL, Haile RW, Stovall M, Boice JD, Jr., Shore RE, Langholz B, et al. Radiation exposure, the ATM Gene, and contralateral breast cancer in the women's environmental cancer and radiation epidemiology study. Journal of the National Cancer Institute. 2010;102(7):475-83.

26. Renwick A, Thompson D, Seal S, Kelly P, Chagtai T, Ahmed M, et al. ATM mutations that cause ataxia-telangiectasia are breast cancer susceptibility alleles. Nature genetics. 2006;38(8):873-5.

27. Guran S, Safali M. A case of neurofibromatosis and breast cancer: loss of heterozygosity of NF1 in breast cancer. Cancer genetics and cytogenetics. 2005;156(1):86-8.

28. Madanikia SA, Bergner A, Ye X, Blakeley JO. Increased risk of breast cancer in women with NF1. American journal of medical genetics Part A. 2012;158A(12):3056-60.

29. Seminog OO, Goldacre MJ. Age-specific risk of breast cancer in women with neurofibromatosis type 1. British journal of cancer. 2015;112(9):1546-8.

30. Boardman LA, Thibodeau SN, Schaid DJ, Lindor NM, McDonnell SK, Burgart LJ, et al. Increased risk for cancer in patients with the Peutz-Jeghers syndrome. Annals of internal medicine. 1998;128(11):896-9.

31. Hemminki A, Markie D, Tomlinson I, Avizienyte E, Roth S, Loukola A, et al. A serine/threonine kinase gene defective in Peutz-Jeghers syndrome. Nature. 1998;391(6663):184-7.

32. Jenne DE, Reimann H, Nezu J, Friedel W, Loff S, Jeschke R, et al. Peutz-Jeghers syndrome is caused by mutations in a novel serine threonine kinase. Nature genetics. 1998;18(1):38-43.

33. Kleibl Z, Kristensen VN. Women at high risk of breast cancer: Molecular characteristics, clinical presentation and management. Breast. 2016;28:136-44.

34. Antoniou AC, Foulkes WD, Tischkowitz M. Breast-cancer risk in families with mutations in PALB2. The New England journal of medicine. 2014;371(17):1651-2.

35. Foo TK, Tischkowitz M, Simhadri S, Boshari T, Zayed N, Burke KA, et al. Compromised BRCA1-PALB2 interaction is associated with breast cancer risk. Oncogene. 2017;36(29):4161-70.

36. Rahman N, Seal S, Thompson D, Kelly P, Renwick A, Elliott A, et al. PALB2, which encodes a BRCA2-interacting protein, is a breast cancer susceptibility gene. Nature genetics. 2007;39(2):165-7.

37. Zhang F, Fan Q, Ren K, Andreassen PR. PALB2 functionally connects the breast cancer susceptibility proteins BRCA1 and BRCA2. Molecular cancer research : MCR. 2009;7(7):1110-8.

38. Moyer CL, Ivanovich J, Gillespie JL, Doberstein R, Radke MR, Richardson ME, et al. Rare BRIP1 Missense Alleles Confer Risk for Ovarian and Breast Cancer. Cancer research. 2020;80(4):857-67.

39. Seal S, Thompson D, Renwick A, Elliott A, Kelly P, Barfoot R, et al. Truncating mutations in the Fanconi anemia J gene BRIP1 are low-penetrance breast cancer susceptibility alleles. Nature genetics. 2006;38(11):1239-41.

40. Bagherzadeh M, Szymiczek A, Donenberg T, Butler R, Hurley J, Narod SA, et al. Association of RAD51C germline mutations with breast cancer among Bahamians. Breast cancer research and treatment. 2020;184(2):649-51.

41. Yang X, Song H, Leslie G, Engel C, Hahnen E, Auber B, et al. Ovarian and Breast Cancer Risks Associated With Pathogenic Variants in RAD51C and RAD51D. Journal of the National Cancer Institute. 2020;112(12):1242-50.

42. Konstanta I, Fostira F, Apostolou P, Stratikos E, Kalfakakou D, Pampanos A, et al. Contribution of RAD51D germline mutations in breast and ovarian cancer in Greece. Journal of human genetics. 2018;63(11):1149-58.

43. Deckers M, van Dinther M, Buijs J, Que I, Lowik C, van der Pluijm G, et al. The tumor suppressor Smad4 is required for transforming growth factor beta-induced epithelial to mesenchymal transition and bone metastasis of breast cancer cells. Cancer research. 2006;66(4):2202-9.

44. Stuelten CH, Buck MB, Dippon J, Roberts AB, Fritz P, Knabbe C. Smad4-expression is decreased in breast cancer tissues: a retrospective study. BMC cancer. 2006;6:25.

45. Wu L, Wu Y, Gathings B, Wan M, Li X, Grizzle W, et al. Smad4 as a transcription corepressor for estrogen receptor alpha. The Journal of biological chemistry. 2003;278(17):15192-200.

46. Zhong D, Morikawa A, Guo L, Colpaert C, Xiong L, Nassar A, et al. Homozygous deletion of SMAD4 in breast cancer cell lines and invasive ductal carcinomas. Cancer biology & therapy. 2006;5(6):601-7.

47. Easton DF. The inherited component of cancer. British medical bulletin. 1994;50(3):527-35.

48. Zhang G, Zeng Y, Liu Z, Wei W. Significant association between Nijmegen breakage syndrome 1 657del5 polymorphism and breast cancer risk. Tumour biology : the journal of the International Society for Oncodevelopmental Biology and Medicine. 2013;34(5):2753-7.

49. Rennert G, Lejbkowicz F, Cohen I, Pinchev M, Rennert HS, Barnett-Griness O. MutYH mutation carriers have increased breast cancer risk. Cancer. 2012;118(8):1989-93.

50. Rizzolo P, Silvestri V, Bucalo A, Zelli V, Valentini V, Catucci I, et al. Contribution of MUTYH Variants to Male Breast Cancer Risk: Results From a Multicenter Study in Italy. Frontiers in oncology. 2018;8:583.

51. Thibodeau ML, Zhao EY, Reisle C, Ch'ng C, Wong HL, Shen Y, et al. Base excision repair deficiency signatures implicate germline and somatic MUTYH aberrations in pancreatic ductal adenocarcinoma and breast cancer oncogenesis. Cold Spring Harbor molecular case studies. 2019;5(2).

52. Pharoah PD, Guilford P, Caldas C, International Gastric Cancer Linkage C. Incidence of gastric cancer and breast cancer in CDH1 (E-cadherin) mutation carriers from hereditary diffuse gastric cancer families. Gastroenterology. 2001;121(6):1348-53.

53. Curia MC, De Iure S, De Lellis L, Veschi S, Mammarella S, White MJ, et al. Increased variance in germline allele-specific expression of APC associates with colorectal cancer. Gastroenterology. 2012;142(1):71-7 e1.

54. Mukherjee N, Bhattacharya N, Sinha S, Alam N, Chakravarti R, Roychoudhury S, et al. Association of APC and MCC polymorphisms with increased breast cancer risk in an Indian population. The International journal of biological markers. 2011;26(1):43-9.

55. Debniak T, Cybulski C, Gorski B, Huzarski T, Byrski T, Gronwald J, et al. CDKN2A-positive breast cancers in young women from Poland. Breast cancer research and treatment. 2007;103(3):355-9.

56. Bera A, Russ E, Manoharan MS, Eidelman O, Eklund M, Hueman M, et al. Proteomic Analysis of Inflammatory Biomarkers Associated With Breast Cancer Recurrence. Military medicine. 2020;185(Suppl 1):669-75.

57. Valo I, Raro P, Boissard A, Maarouf A, Jezequel P, Verriele V, et al. OLFM4 Expression in Ductal Carcinoma In Situ and in Invasive Breast Cancer Cohorts by a SWATH-Based Proteomic Approach. Proteomics. 2019;19(21-22):e1800446.

58. Zeidan B, Manousopoulou A, Garay-Baquero DJ, White CH, Larkin SET, Potter KN, et al. Increased circulating resistin levels in early-onset breast cancer patients of normal body mass index correlate with lymph node negative involvement and longer disease free survival: a multi-center POSH cohort serum proteomics study. Breast cancer research : BCR. 2018;20(1):19.

59. Shao B, Tian Z, Ding H, Wang Q, Song G, Di L, et al. Plasma CAMK2A predicts chemotherapy resistance in metastatic triple negative breast cancer. International journal of clinical and experimental pathology. 2018;11(2):650-63.

60. Correa S, Panis C, Binato R, Herrera AC, Pizzatti L, Abdelhay E. Identifying potential markers in Breast Cancer subtypes using plasma label-free proteomics. Journal of proteomics. 2017;151:33-42.

61. Lobo MD, Moreno FB, Souza GH, Verde SM, Moreira RA, Monteiro-Moreira AC. Label-Free Proteome Analysis of Plasma from Patients with Breast Cancer: Stage-Specific Protein Expression. Frontiers in oncology. 2017;7:14.

62. Fu-Jun L, Shao-Hua J, Xiao-Fang S. Differential proteomic analysis of pathway biomarkers in human breast cancer by integrated bioinformatics. Oncology letters. 2012;4(5):1097-103.

63. Gajbhiye A, Dabhi R, Taunk K, Vannuruswamy G, RoyChoudhury S, Adhav R, et al. Urinary proteome alterations in HER2 enriched breast cancer revealed by multipronged quantitative proteomics. Proteomics. 2016;16(17):2403-18.

64. Scumaci D, Tamme L, Fiumara CV, Pappaianni G, Concolino A, Leone E, et al. Plasma Proteomic Profiling in Hereditary Breast Cancer Reveals a BRCA1-Specific Signature: Diagnostic and Functional Implications. PloS one. 2015;10(6):e0129762.

65. Lopez-Arias E, Aguilar-Lemarroy A, Felipe Jave-Suarez L, Morgan-Villela G, Mariscal-Ramirez I, Martinez-Velazquez M, et al. Alpha 1-antitrypsin: a novel tumor-associated antigen identified in patients with early-stage breast cancer. Electrophoresis. 2012;33(14):2130-7.

66. Kurono S, Kaneko Y, Matsuura N, Oishi H, Noguchi S, Kim SJ, et al. Identification of potential breast cancer markers in nipple discharge by protein profile analysis using two-dimensional nano-liquid chromatography/nanoelectrospray ionization-mass spectrometry. Proteomics Clinical applications. 2016;10(5):605-13.

67. Lee CS, Taib NA, Ashrafzadeh A, Fadzli F, Harun F, Rahmat K, et al. Unmasking Heavily O-Glycosylated Serum Proteins Using Perchloric Acid: Identification of Serum Proteoglycan 4 and Protease C1 Inhibitor as Molecular Indicators for Screening of Breast Cancer. PloS one. 2016;11(2):e0149551.

68. Beretov J, Wasinger VC, Millar EK, Schwartz P, Graham PH, Li Y. Proteomic Analysis of Urine to Identify Breast Cancer Biomarker Candidates Using a Label-Free LC-MS/MS Approach. PloS one. 2015;10(11):e0141876.

69. Lee HB, Kang UB, Moon HG, Lee J, Lee KM, Yi M, et al. Development and Validation of a Novel Plasma Protein Signature for Breast Cancer Diagnosis by Using Multiple Reaction Monitoring-based Mass Spectrometry. Anticancer research. 2015;35(11):6271-9.

70. Chung L, Moore K, Phillips L, Boyle FM, Marsh DJ, Baxter RC. Novel serum protein biomarker panel revealed by mass spectrometry and its prognostic value in breast cancer. Breast cancer research : BCR. 2014;16(3):R63.

71. Fan Y, Wang J, Yang Y, Liu Q, Fan Y, Yu J, et al. Detection and identification of potential biomarkers of breast cancer. Journal of cancer research and clinical oncology. 2010;136(8):1243-54.

72. Fernandez-Grijalva AL, Aguilar-Lemarroy A, Jave-Suarez LF, Gutierrez-Ortega A, Godinez-Melgoza PA, Herrera-Rodriguez SE, et al. Alpha 2HS-glycoprotein, a tumor-associated antigen (TAA) detected in Mexican patients with early-stage breast cancer. Journal of proteomics. 2015;112:301-12.

73. Hyung SW, Lee MY, Yu JH, Shin B, Jung HJ, Park JM, et al. A serum protein profile predictive of the resistance to neoadjuvant chemotherapy in advanced breast cancers. Molecular & cellular proteomics : MCP. 2011;10(10):M111 011023.

74. Boccardo F, Rubagotti A, Nuzzo PV, Argellati F, Savarino G, Romano P, et al. Matrix-assisted laser desorption/ionisation (MALDI) TOF analysis identifies serum angiotensin II concentrations as a strong predictor of all-cause and breast cancer (BCa)-specific mortality following breast surgery. International journal of cancer. 2015;137(10):2394-402.

75. Zeidan B, Jackson TR, Larkin SE, Cutress RI, Coulton GR, Ashton-Key M, et al. Annexin A3 is a mammary marker and a potential neoplastic breast cell therapeutic target. Oncotarget. 2015;6(25):21421-7.

76. Brauer HA, D'Arcy M, Libby TE, Thompson HJ, Yasui YY, Hamajima N, et al. Dermcidin expression is associated with disease progression and survival among breast cancer patients. Breast cancer research and treatment. 2014;144(2):299-306.

77. Orlandi R, De Bortoli M, Ciniselli CM, Vaghi E, Caccia D, Garrisi V, et al. Hepcidin and ferritin blood level as noninvasive tools for predicting breast cancer. Annals of oncology : official journal of the European Society for Medical Oncology. 2014;25(2):352-7.

78. Panis C, Pizzatti L, Herrera AC, Cecchini R, Abdelhay E. Putative circulating markers of the early and advanced stages of breast cancer identified by high-resolution label-free proteomics. Cancer letters. 2013;330(1):57-66.

79. He W, Tong Y, Wang Y, Liu J, Luo G, Wu J, et al. Serum soluble CD14 is a potential prognostic indicator of recurrence of human breast invasive ductal carcinoma with Her2-enriched subtype. PloS one. 2013;8(9):e75366.

80. Suh EJ, Kabir MH, Kang UB, Lee JW, Yu J, Noh DY, et al. Comparative profiling of plasma proteome from breast cancer patients reveals thrombospondin-1 and BRWD3 as serological biomarkers. Experimental & molecular medicine. 2012;44(1):36-44.

81. Fry SA, Sinclair J, Timms JF, Leathem AJ, Dwek MV. A targeted glycoproteomic approach identifies cadherin-5 as a novel biomarker of metastatic breast cancer. Cancer letters. 2013;328(2):335-44.

82. Lacombe J, Mange A, Jarlier M, Bascoul-Mollevi C, Rouanet P, Lamy PJ, et al. Identification and validation of new autoantibodies for the diagnosis of DCIS and node negative early-stage breast cancers. International journal of cancer. 2013;132(5):1105-13.

83. Pitteri SJ, Amon LM, Busald Buson T, Zhang Y, Johnson MM, Chin A, et al. Detection of elevated plasma levels of epidermal growth factor receptor before breast cancer diagnosis among hormone therapy users. Cancer research. 2010;70(21):8598-606.

84. Hebert JD, Myers SA, Naba A, Abbruzzese G, Lamar JM, Carr SA, et al. Proteomic Profiling of the ECM of Xenograft Breast Cancer Metastases in Different Organs Reveals Distinct Metastatic Niches. Cancer research. 2020;80(7):1475-85.

85. Chen Z, Yan X, Li K, Ling Y, Kang H. Stromal fibroblast-derived MFAP5 promotes the invasion and migration of breast cancer cells via Notch1/slug signaling. Clinical & translational oncology : official publication of the Federation of Spanish Oncology Societies and of the National Cancer Institute of Mexico. 2020;22(4):522-31.

86. Warmoes M, Lam SW, van der Groep P, Jaspers JE, Smolders YH, de Boer L, et al. Secretome proteomics reveals candidate non-invasive biomarkers of BRCA1 deficiency in breast cancer. Oncotarget. 2016;7(39):63537-48.

87. Lee JE, Moon PG, Cho YE, Kim YB, Kim IS, Park H, et al. Identification of EDIL3 on extracellular vesicles involved in breast cancer cell invasion. Journal of proteomics. 2016;131:17-28.

88. Naba A, Clauser KR, Lamar JM, Carr SA, Hynes RO. Extracellular matrix signatures of human mammary carcinoma identify novel metastasis promoters. eLife. 2014;3:e01308.

89. Jeon YR, Kim SY, Lee EJ, Kim YN, Noh DY, Park SY, et al. Identification of annexin II as a novel secretory biomarker for breast cancer. Proteomics. 2013;13(21):3145-56.

90. Whelan SA, He J, Lu M, Souda P, Saxton RE, Faull KF, et al. Mass spectrometry (LC-MS/MS) identified proteomic biosignatures of breast cancer in proximal fluid. Journal of proteome research. 2012;11(10):5034-45.

91. Pateetin P, Pisitkun T, McGowan E, Boonyaratanakornkit V. Differential quantitative proteomics reveals key proteins related to phenotypic changes of breast cancer cells expressing progesterone receptor A. The Journal of steroid biochemistry and molecular biology. 2020;198:105560.

92. Yang YL, Zhang Y, Li DD, Zhang FL, Liu HY, Liao XH, et al. RNF144A functions as a tumor suppressor in breast cancer through ubiquitin ligase activity-dependent regulation of stability and oncogenic functions of HSPA2. Cell death and differentiation. 2020;27(3):1105-18.

93. Koh EY, You JE, Jung SH, Kim PH. Biological Functions and Identification of Novel Biomarker Expressed on the Surface of Breast Cancer-Derived Cancer Stem Cells via Proteomic Analysis. Molecules and cells. 2020;43(4):384-96.

94. Kosok M, Alli-Shaik A, Bay BH, Gunaratne J. Comprehensive Proteomic Characterization Reveals Subclass-Specific Molecular Aberrations within Triple-negative Breast Cancer. iScience. 2020;23(2):100868.

95. Blazquez R, Rietkotter E, Wenske B, Wlochowitz D, Sparrer D, Vollmer E, et al. LEF1 supports metastatic brain colonization by regulating glutathione metabolism and increasing ROS resistance in breast cancer. International journal of cancer. 2020;146(11):3170-83.

96. Askeland C, Wik E, Finne K, Birkeland E, Arnes JB, Collett K, et al. Stathmin expression associates with vascular and immune responses in aggressive breast cancer subgroups. Scientific reports. 2020;10(1):2914.

97. Prochazkova I, Lenco J, Fucikova A, Dresler J, Capkova L, Hrstka R, et al. Targeted proteomics driven verification of biomarker candidates associated with breast cancer aggressiveness. Biochimica et biophysica acta Proteins and proteomics. 2017;1865(5):488-98.

98. Zeng L, Deng X, Zhong J, Yuan L, Tao X, Zhang S, et al. Prognostic value of biomarkers EpCAM and alphaB-crystallin associated with lymphatic metastasis in breast cancer by iTRAQ analysis. BMC cancer. 2019;19(1):831.

99. Silvestrini VC, Thome CH, Albuquerque D, de Souza Palma C, Ferreira GA, Lanfredi GP, et al. Proteomics analysis reveals the role of ubiquitin specific protease (USP47) in Epithelial to Mesenchymal Transition (EMT) induced by TGFbeta2 in breast cells. Journal of proteomics. 2020;219:103734.

100. Rojas LK, Trilla-Fuertes L, Gamez-Pozo A, Chiva C, Sepulveda J, Manso L, et al. Proteomics characterisation of central nervous system metastasis biomarkers in triple negative breast cancer. Ecancermedicalscience. 2019;13:891.

101. Liu NQ, Stingl C, Look MP, Smid M, Braakman RB, De Marchi T, et al. Comparative proteome analysis revealing an 11-protein signature for aggressive triple-negative breast cancer. Journal of the National Cancer Institute. 2014;106(2):djt376.

102. Yoneten KK, Kasap M, Akpinar G, Gunes A, Gurel B, Utkan NZ. Comparative Proteome Analysis of Breast Cancer Tissues Highlights the Importance of Glycerol-3-phosphate Dehydrogenase 1 and Monoacylglycerol Lipase in Breast Cancer Metabolism. Cancer genomics & proteomics. 2019;16(5):377-97.

103. Brown JE, Westbrook JA, Wood SL. Dedicator of Cytokinesis 4: A Potential Prognostic and Predictive Biomarker Within the Metastatic Spread of Breast Cancer to Bone. Cancer informatics. 2019;18:1176935119866842.

104. Westbrook JA, Wood SL, Cairns DA, McMahon K, Gahlaut R, Thygesen H, et al. Identification and validation of DOCK4 as a potential biomarker for risk of bone metastasis development in patients with early breast cancer. The Journal of pathology. 2019;247(3):381-91.

105. Cordero A, Kanojia D, Miska J, Panek WK, Xiao A, Han Y, et al. FABP7 is a key metabolic regulator in HER2+ breast cancer brain metastasis. Oncogene. 2019;38(37):6445-60.

106. Faktor J, Knopfova L, Lapcik P, Janacova L, Paralova V, Bouchalova P, et al. Proteomics Identification and Validation of Desmocollin-1 and Catechol-O-Methyltransferase as Proteins Associated with Breast Cancer Cell Migration and Metastasis. Proteomics. 2019;19(21-22):e1900073.

107. Uretmen Kagiali ZC, Sanal E, Karayel O, Polat AN, Saatci O, Ersan PG, et al. Systems-level Analysis Reveals Multiple Modulators of Epithelial-mesenchymal Transition and Identifies DNAJB4 and CD81 as Novel Metastasis Inducers in Breast Cancer. Molecular & cellular proteomics : MCP. 2019;18(9):1756-71.

108. de Boer HR, Pool M, Joosten E, Everts M, Samplonius DF, Helfrich W, et al. Quantitative proteomics analysis identifies MUC1 as an effect sensor of EGFR inhibition. Oncogene. 2019;38(9):1477-88.

109. Peng W, Zhang Y, Zhu R, Mechref Y. Comparative membrane proteomics analyses of breast cancer cell lines to understand the molecular mechanism of breast cancer brain metastasis. Electrophoresis. 2017;38(17):2124-34.

110. Soudy R, Etayash H, Bahadorani K, Lavasanifar A, Kaur K. Breast Cancer Targeting Peptide Binds Keratin 1: A New Molecular Marker for Targeted Drug Delivery to Breast Cancer. Molecular pharmaceutics. 2017;14(3):593-604.

111. Shaheed SU, Rustogi N, Scally A, Wilson J, Thygesen H, Loizidou MA, et al. Identification of stage-specific breast markers using quantitative proteomics. Journal of proteome research. 2013;12(12):5696-708.

112. Pedersen MH, Hood BL, Beck HC, Conrads TP, Ditzel HJ, Leth-Larsen R. Downregulation of antigen presentation-associated pathway proteins is linked to poor outcome in triple-negative breast cancer patient tumors. Oncoimmunology. 2017;6(5):e1305531.

113. Mertins P, Mani DR, Ruggles KV, Gillette MA, Clauser KR, Wang P, et al. Proteogenomics connects somatic mutations to signalling in breast cancer. Nature. 2016;534(7605):55-62.

114. Dvorakova M, Jerabkova J, Prochazkova I, Lenco J, Nenutil R, Bouchal P. Transgelin is upregulated in stromal cells of lymph node positive breast cancer. Journal of proteomics. 2016;132:103-11.

115. Shin J, Kim G, Lee JW, Lee JE, Kim YS, Yu JH, et al. Identification of ganglioside GM2 activator playing a role in cancer cell migration through proteomic analysis of breast cancer secretomes. Cancer science. 2016;107(6):828-35.

116. Bouchal P, Dvorakova M, Roumeliotis T, Bortlicek Z, Ihnatova I, Prochazkova I, et al. Combined Proteomics and Transcriptomics Identifies Carboxypeptidase B1 and Nuclear Factor kappaB (NF-kappaB) Associated Proteins as Putative Biomarkers of Metastasis in Low Grade Breast Cancer. Molecular & cellular proteomics : MCP. 2015;14(7):1814-30.

117. Calderon-Gonzalez KG, Valero Rustarazo ML, Labra-Barrios ML, Bazan-Mendez CI, Tavera-Tapia A, Herrera-Aguirre ME, et al. Determination of the protein expression profiles of breast cancer cell lines by quantitative proteomics using iTRAQ labelling and tandem mass spectrometry. Journal of proteomics. 2015;124:50-78.

118. Dun MD, Chalkley RJ, Faulkner S, Keene S, Avery-Kiejda KA, Scott RJ, et al. Proteotranscriptomic Profiling of 231-BR Breast Cancer Cells: Identification of Potential Biomarkers and Therapeutic Targets for Brain Metastasis. Molecular & cellular proteomics : MCP. 2015;14(9):2316-30.

119. Li LD, Sun HF, Liu XX, Gao SP, Jiang HL, Hu X, et al. Down-Regulation of NDUFB9 Promotes Breast Cancer Cell Proliferation, Metastasis by Mediating Mitochondrial Metabolism. PloS one. 2015;10(12):e0144441.

120. Jiang HL, Sun HF, Gao SP, Li LD, Hu X, Wu J, et al. Loss of RAB1B promotes triple-negative breast cancer metastasis by activating TGF-beta/SMAD signaling. Oncotarget. 2015;6(18):16352-65.

121. Johansson HJ, Sanchez BC, Forshed J, Stal O, Fohlin H, Lewensohn R, et al. Proteomics profiling identify CAPS as a potential predictive marker of tamoxifen resistance in estrogen receptor positive breast cancer. Clinical proteomics. 2015;12(1):8.

122. Lund RR, Leth-Larsen R, Caterino TD, Terp MG, Nissen J, Laenkholm AV, et al. NADH-Cytochrome b5 Reductase 3 Promotes Colonization and Metastasis Formation and Is a Prognostic Marker of Disease-Free and Overall Survival in Estrogen Receptor-Negative Breast Cancer. Molecular & cellular proteomics : MCP. 2015;14(11):2988-99.

123. Sato M, Matsubara T, Adachi J, Hashimoto Y, Fukamizu K, Kishida M, et al. Differential Proteome Analysis Identifies TGF-beta-Related Pro-Metastatic Proteins in a 4T1 Murine Breast Cancer Model. PloS one. 2015;10(5):e0126483.

124. Damaghi M, Tafreshi NK, Lloyd MC, Sprung R, Estrella V, Wojtkowiak JW, et al. Chronic acidosis in the tumour microenvironment selects for overexpression of LAMP2 in the plasma membrane. Nature communications. 2015;6:8752.

125. Muniz Lino MA, Palacios-Rodriguez Y, Rodriguez-Cuevas S, Bautista-Pina V, Marchat LA, Ruiz-Garcia E, et al. Comparative proteomic profiling of triple-negative breast cancer reveals that up-regulation of RhoGDI-2 is associated to the inhibition of caspase 3 and caspase 9. Journal of proteomics. 2014;111:198-211.

126. Linge A, Maurya P, Friedrich K, Baretton GB, Kelly S, Henry M, et al. Identification and functional validation of RAD23B as a potential protein in human breast cancer progression. Journal of proteome research. 2014;13(7):3212-22.

127. Jezequel P, Campion L, Spyratos F, Loussouarn D, Campone M, Guerin-Charbonnel C, et al. Validation of tumor-associated macrophage ferritin light chain as a prognostic biomarker in node-negative breast cancer tumors: A multicentric 2004 national PHRC study. International journal of cancer. 2012;131(2):426-37.

128. Kanojia D, Zhou W, Zhang J, Jie C, Lo PK, Wang Q, et al. Proteomic profiling of cancer stem cells derived from primary tumors of HER2/Neu transgenic mice. Proteomics. 2012;12(22):3407-15.

129. Cain JW, Hauptschein RS, Stewart JK, Bagci T, Sahagian GG, Jay DG. Identification of CD44 as a surface biomarker for drug resistance by surface proteome signature technology. Molecular cancer research : MCR. 2011;9(5):637-47.

130. Rust S, Guillard S, Sachsenmeier K, Hay C, Davidson M, Karlsson A, et al. Combining phenotypic and proteomic approaches to identify membrane targets in a 'triple negative' breast cancer cell type. Molecular cancer. 2013;12:11.

131. Morrison BJ, Hastie ML, Grewal YS, Bruce ZC, Schmidt C, Reynolds BA, et al. Proteomic comparison of mcf-7 tumoursphere and monolayer cultures. PloS one. 2012;7(12):e52692.

132. Greenwood C, Metodieva G, Al-Janabi K, Lausen B, Alldridge L, Leng L, et al. Stat1 and CD74 overexpression is co-dependent and linked to increased invasion and lymph node metastasis in triple-negative breast cancer. Journal of proteomics. 2012;75(10):3031-40.

133. Metodieva G, Nogueira-de-Souza NC, Greenwood C, Al-Janabi K, Leng L, Bucala R, et al. CD74-dependent deregulation of the tumor suppressor scribble in human epithelial and breast cancer cells. Neoplasia. 2013;15(6):660-8.

134. Kabbage M, Trimeche M, Bergaoui S, Hammann P, Kuhn L, Hamrita B, et al. Calreticulin expression in infiltrating ductal breast carcinomas: relationships with disease progression and humoral immune responses. Tumour biology : the journal of the International Society for Oncodevelopmental Biology and Medicine. 2013;34(2):1177-88.

135. Song MN, Moon PG, Lee JE, Na M, Kang W, Chae YS, et al. Proteomic analysis of breast cancer tissues to identify biomarker candidates by gel-assisted digestion and label-free quantification methods using LC-MS/MS. Archives of pharmacal research. 2012;35(10):1839-47.

136. Fonseca-Sanchez MA, Rodriguez Cuevas S, Mendoza-Hernandez G, Bautista-Pina V, Arechaga Ocampo E, Hidalgo Miranda A, et al. Breast cancer proteomics reveals a positive correlation between glyoxalase 1 expression and high tumor grade. International journal of oncology. 2012;41(2):670-80.

137. Geiger T, Madden SF, Gallagher WM, Cox J, Mann M. Proteomic portrait of human breast cancer progression identifies novel prognostic markers. Cancer research. 2012;72(9):2428-39.

138. Pendharkar N, Gajbhiye A, Taunk K, RoyChoudhury S, Dhali S, Seal S, et al. Quantitative tissue proteomic investigation of invasive ductal carcinoma of breast with luminal B HER2 positive and HER2 enriched subtypes towards potential diagnostic and therapeutic biomarkers. Journal of proteomics. 2016;132:112-30.

139. Cawthorn TR, Moreno JC, Dharsee M, Tran-Thanh D, Ackloo S, Zhu PH, et al. Proteomic analyses reveal high expression of decorin and endoplasmin (HSP90B1) are associated with breast cancer metastasis and decreased survival. PloS one. 2012;7(2):e30992.

140. Wang D, Huang J, Hu Z. RNA helicase DDX5 regulates microRNA expression and contributes to cytoskeletal reorganization in basal breast cancer cells. Molecular & cellular proteomics : MCP. 2012;11(2):M111 011932.

141. Terp MG, Lund RR, Jensen ON, Leth-Larsen R, Ditzel HJ. Identification of markers associated with highly aggressive metastatic phenotypes using quantitative comparative proteomics. Cancer genomics & proteomics. 2012;9(5):265-73.

142. Pan J, Sun LC, Tao YF, Zhou Z, Du XL, Peng L, et al. ATP synthase ecto-alpha-subunit: a novel therapeutic target for breast cancer. Journal of translational medicine. 2011;9:211.

143. Wang ZC, E D, Batu DL, Saixi YL, Zhang B, Ren LQ. 2D-DIGE proteomic analysis of changes in estrogen/progesterone-induced rat breast hyperplasia upon treatment with the Mongolian remedy RuXian-I. Molecules. 2011;16(4):3048-65.

144. Qiu Y, Zhou B, Su M, Baxter S, Zheng X, Zhao X, et al. Mass spectrometry-based quantitative metabolomics revealed a distinct lipid profile in breast cancer patients. International journal of molecular sciences. 2013;14(4):8047-61.

145. Burton C, Ma Y. Current Trends in Cancer Biomarker Discovery Using Urinary Metabolomics: Achievements and New Challenges. Current medicinal chemistry. 2019;26(1):5-28.

146. Nam H, Chung BC, Kim Y, Lee K, Lee D. Combining tissue transcriptomics and urine metabolomics for breast cancer biomarker identification. Bioinformatics. 2009;25(23):3151-7.

147. Slupsky CM, Steed H, Wells TH, Dabbs K, Schepansky A, Capstick V, et al. Urine metabolite analysis offers potential early diagnosis of ovarian and breast cancers. Clinical cancer research : an official journal of the American Association for Cancer Research. 2010;16(23):5835-41.

148. Woo HM, Kim KM, Choi MH, Jung BH, Lee J, Kong G, et al. Mass spectrometry based metabolomic approaches in urinary biomarker study of women's cancers. Clinica chimica acta; international journal of clinical chemistry. 2009;400(1-2):63-9.

149. Asiago VM, Alvarado LZ, Shanaiah N, Gowda GA, Owusu-Sarfo K, Ballas RA, et al. Early detection of recurrent breast cancer using metabolite profiling. Cancer research. 2010;70(21):8309-18.

150. Jobard E, Pontoizeau C, Blaise BJ, Bachelot T, Elena-Herrmann B, Tredan O. A serum nuclear magnetic resonance-based metabolomic signature of advanced metastatic human breast cancer. Cancer letters. 2014;343(1):33-41.

151. Zhong L, Cheng F, Lu X, Duan Y, Wang X. Untargeted saliva metabonomics study of breast cancer based on ultra performance liquid chromatography coupled to mass spectrometry with HILIC and RPLC separations. Talanta. 2016;158:351-60.

152. Fan Y, Zhou X, Xia TS, Chen Z, Li J, Liu Q, et al. Human plasma metabolomics for identifying differential metabolites and predicting molecular subtypes of breast cancer. Oncotarget. 2016;7(9):9925-38.

153. Oakman C, Tenori L, Claudino WM, Cappadona S, Nepi S, Battaglia A, et al. Identification of a serum-detectable metabolomic fingerprint potentially correlated with the presence of micrometastatic disease in early breast cancer patients at varying risks of disease relapse by traditional prognostic methods. Annals of oncology : official journal of the European Society for Medical Oncology. 2011;22(6):1295-301.

154. Wang Q, Sun T, Cao Y, Gao P, Dong J, Fang Y, et al. A dried blood spot mass spectrometry metabolomic approach for rapid breast cancer detection. OncoTargets and therapy. 2016;9:1389-98.

155. Cala M, Aldana J, Sanchez J, Guio J, Meesters RJW. Urinary metabolite and lipid alterations in Colombian Hispanic women with breast cancer: A pilot study. Journal of pharmaceutical and biomedical analysis. 2018;152:234-41.

156. Huang S, Chong N, Lewis NE, Jia W, Xie G, Garmire LX. Novel personalized pathway-based metabolomics models reveal key metabolic pathways for breast cancer diagnosis. Genome medicine. 2016;8(1):34.

157. Chen Y, Zhang R, Song Y, He J, Sun J, Bai J, et al. RRLC-MS/MS-based metabonomics combined with in-depth analysis of metabolic correlation network: finding potential biomarkers for breast cancer. The Analyst. 2009;134(10):2003-11.

158. Jove M, Collado R, Quiles JL, Ramirez-Tortosa MC, Sol J, Ruiz-Sanjuan M, et al. A plasma metabolomic signature discloses human breast cancer. Oncotarget. 2017;8(12):19522-33.

159. Lecuyer L, Victor Bala A, Deschasaux M, Bouchemal N, Nawfal Triba M, Vasson MP, et al. NMR metabolomic signatures reveal predictive plasma metabolites associated with long-term risk of developing breast cancer. International journal of epidemiology. 2018;47(2):484-94.

160. Lv W, Yang T. Identification of possible biomarkers for breast cancer from free fatty acid profiles determined by GC-MS and multivariate statistical analysis. Clinical biochemistry. 2012;45(1-2):127-33.

161. Takayama T, Tsutsui H, Shimizu I, Toyama T, Yoshimoto N, Endo Y, et al. Diagnostic approach to breast cancer patients based on target metabolomics in saliva by liquid chromatography with tandem mass spectrometry. Clinica chimica acta; international journal of clinical chemistry. 2016;452:18-26.

162. Tsutsui H, Mochizuki T, Inoue K, Toyama T, Yoshimoto N, Endo Y, et al. High-throughput LC-MS/MS based simultaneous determination of polyamines including N-acetylated forms in human saliva and the diagnostic approach to breast cancer patients. Analytical chemistry. 2013;85(24):11835-42.

163. Franky Dhaval S, Shilin Nandubhai S, Pankaj Manubhai S, Patel HR, Prabhudas Shankerbhai P. Significance of alterations in plasma lipid profile levels in breast cancer. Integrative cancer therapies. 2008;7(1):33-41.

164. Cavaco C, Pereira JAM, Taunk K, Taware R, Rapole S, Nagarajaram H, et al. Screening of salivary volatiles for putative breast cancer discrimination: an exploratory study involving geographically distant populations. Analytical and bioanalytical chemistry. 2018;410(18):4459-68.

165. Dougan MM, Li Y, Chu LW, Haile RW, Whittemore AS, Han SS, et al. Metabolomic profiles in breast cancer:a pilot case-control study in the breast cancer family registry. BMC cancer. 2018;18(1):532.

166. Roig B, Rodriguez-Balada M, Samino S, Lam EW, Guaita-Esteruelas S, Gomes AR, et al. Metabolomics reveals novel blood plasma biomarkers associated to the BRCA1-mutated phenotype of human breast cancer. Scientific reports. 2017;7(1):17831.

167. Hadi NI, Jamal Q. "OMIC" tumor markers for breast cancer: A review. Pakistan journal of medical sciences. 2015;31(5):1256-62.

168. Giskeodegard GF, Lundgren S, Sitter B, Fjosne HE, Postma G, Buydens LM, et al. Lactate and glycine-potential MR biomarkers of prognosis in estrogen receptor-positive breast cancers. NMR in biomedicine. 2012;25(11):1271-9.

169. Bathen TF, Geurts B, Sitter B, Fjosne HE, Lundgren S, Buydens LM, et al. Feasibility of MR metabolomics for immediate analysis of resection margins during breast cancer surgery. PloS one. 2013;8(4):e61578.

170. Budhu A, Terunuma A, Zhang G, Hussain SP, Ambs S, Wang XW. Metabolic profiles are principally different between cancers of the liver, pancreas and breast. International journal of biological sciences. 2014;10(9):966-72.

171. Li M, Song Y, Cho N, Chang JM, Koo HR, Yi A, et al. An HR-MAS MR metabolomics study on breast tissues obtained with core needle biopsy. PloS one. 2011;6(10):e25563.

172. Sitter B, Lundgren S, Bathen TF, Halgunset J, Fjosne HE, Gribbestad IS. Comparison of HR MAS MR spectroscopic profiles of breast cancer tissue with clinical parameters. NMR in biomedicine. 2006;19(1):30-40.

173. Mimmi MC, Picotti P, Corazza A, Betto E, Pucillo CE, Cesaratto L, et al. High-performance metabolic marker assessment in breast cancer tissue by mass spectrometry. Clinical chemistry and laboratory medicine. 2011;49(2):317-24.

174. Choi JS, Baek HM, Kim S, Kim MJ, Youk JH, Moon HJ, et al. HR-MAS MR spectroscopy of breast cancer tissue obtained with core needle biopsy: correlation with prognostic factors. PloS one. 2012;7(12):e51712.

175. Griffin JL, Shockcor JP. Metabolic profiles of cancer cells. Nature reviews Cancer. 2004;4(7):551-61.

176. Brockmoller SF, Bucher E, Muller BM, Budczies J, Hilvo M, Griffin JL, et al. Integration of metabolomics and expression of glycerol-3-phosphate acyltransferase (GPAM) in breast cancer-link to patient survival, hormone receptor status, and metabolic profiling. Journal of proteome research. 2012;11(2):850-60.

177. Spratlin JL, Serkova NJ, Eckhardt SG. Clinical applications of metabolomics in oncology: a review. Clinical cancer research : an official journal of the American Association for Cancer Research. 2009;15(2):431-40.

178. Cifkova E, Holcapek M, Lisa M, Vrana D, Gatek J, Melichar B. Determination of lipidomic differences between human breast cancer and surrounding normal tissues using HILIC-HPLC/ESI-MS and multivariate data analysis. Analytical and bioanalytical chemistry. 2015;407(3):991-1002.

179. Mimmi MC, Finato N, Pizzolato G, Beltrami CA, Fogolari F, Corazza A, et al. Absolute quantification of choline-related biomarkers in breast cancer biopsies by liquid chromatography electrospray ionization mass spectrometry. Analytical cellular pathology. 2013;36(3-4):71-83.

180. Beckonert O, Monnerjahn J, Bonk U, Leibfritz D. Visualizing metabolic changes in breast-cancer tissue using 1H-NMR spectroscopy and self-organizing maps. NMR in biomedicine. 2003;16(1):1-11.

181. Doria ML, Cotrim Z, Macedo B, Simoes C, Domingues P, Helguero L, et al. Lipidomic approach to identify patterns in phospholipid profiles and define class differences in mammary epithelial and breast cancer cells. Breast cancer research and treatment. 2012;133(2):635-48.

182. Budczies J, Brockmoller SF, Muller BM, Barupal DK, Richter-Ehrenstein C, Kleine-Tebbe A, et al. Comparative metabolomics of estrogen receptor positive and estrogen receptor negative breast cancer: alterations in glutamine and beta-alanine metabolism. Journal of proteomics. 2013;94:279-88.

183. Budczies J, Denkert C, Muller BM, Brockmoller SF, Klauschen F, Gyorffy B, et al. Remodeling of central metabolism in invasive breast cancer compared to normal breast tissue - a GC-TOFMS based metabolomics study. BMC genomics. 2012;13:334.

184. Willmann L, Schlimpert M, Hirschfeld M, Erbes T, Neubauer H, Stickeler E, et al. Alterations of the exo- and endometabolite profiles in breast cancer cell lines: A mass spectrometry-based metabolomics approach. Analytica chimica acta. 2016;925:34-42.

185. Silva CL, Perestrelo R, Silva P, Tomas H, Camara JS. Volatile metabolomic signature of human breast cancer cell lines. Scientific reports. 2017;7:43969.

186. Schiffmann S, Sandner J, Birod K, Wobst I, Angioni C, Ruckhaberle E, et al. Ceramide synthases and ceramide levels are increased in breast cancer tissue. Carcinogenesis. 2009;30(5):745-52.

187. Kramer A, Green J, Pollard J, Jr., Tugendreich S. Causal analysis approaches in Ingenuity Pathway Analysis. Bioinformatics. 2014;30(4):523-30.
